# The Effects of Astaxanthin Supplementation on Exercise Recovery Biomarkers and Exercise Performance: A Systematic Review and Meta-Analysis

**DOI:** 10.3390/nu18101570

**Published:** 2026-05-15

**Authors:** Shuning Liu, Wenqian Yao, Yan Wei, Samuhaer Azhati, Yutong Wu, Wen Zhong, Pengda Wang, Heping Dai, Kai Zhao, Chang Liu

**Affiliations:** 1 School of Sports Science, Beijing Sport University, Beijing 100084, China; 2023013553@bsu.edu.cn (S.L.); 2024012786@bsu.edu.cn (Y.W.); 2025012597@bsu.edu.cn (W.Z.); 2 School of Journalism and Communication, Beijing Sport University, Beijing 100084, China; 2023013417@bsu.edu.cn; 3 School of Education, Beijing Sport University, Beijing 100084, China; 2024011355@bsu.edu.cn (Y.W.); sam@bsu.edu.cn (S.A.); 4 School of Physical Education, Minzu University of China, Beijing 100074, China; 25301974@muc.edu.cn; 5 China Wushu School, Beijing Sport University, Beijing 100084, China; 17673025508@163.com; 6 China Volleyball Academy, Beijing Sport University, Beijing 100084, China

**Keywords:** astaxanthin, exercise recovery, exercise performance, muscle damage, oxidative stress, meta-analysis

## Abstract

**Background**: Astaxanthin is a lipid-soluble carotenoid with antioxidant and anti-inflammatory properties, but its effects on exercise performance and post-exercise recovery remain uncertain. This systematic review and meta-analysis aimed to evaluate the effects of astaxanthin supplementation on exercise performance and recovery-related biomarkers in healthy participants and athletes. **Methods**: This review followed PRISMA 2020 guidelines. PubMed, Web of Science, Embase, EBSCO, the Cochrane Library, and CNKI were searched from inception to January 2026. Randomized controlled trials comparing oral astaxanthin supplementation with placebo or control were included. Performance outcomes included VO_2_max, time-trial or endurance-related performance, and maximal workload or power output. Recovery-related outcomes included creatine kinase, lactate dehydrogenase, malondialdehyde, interleukin-6, and related biomarkers. Standardized mean differences with 95% confidence intervals were pooled. **Results**: Twenty-four RCTs were included. Astaxanthin significantly reduced creatine kinase levels (SMD = −0.45, 95% CI: −0.83 to −0.07). Lactate dehydrogenase also favored astaxanthin (SMD = −0.93, 95% CI: −1.39 to −0.48), although heterogeneity was substantial. No significant effects were observed for malondialdehyde or interleukin-6. Astaxanthin did not significantly improve VO_2_max, time-trial performance, or maximal workload/power output. **Conclusions**: Current evidence suggests that astaxanthin may be more beneficial for post-exercise recovery than for direct performance enhancement. The most consistent effect was observed for creatine kinase, whereas the LDH finding should be interpreted cautiously. Further well-powered trials with standardized dosing, duration, exercise protocols, and outcome assessments are needed.

## 1. Introduction

Repeated bouts of strenuous exercise can induce substantial metabolic and mechanical stress, particularly when the training load is high, competition schedules are congested, or recovery time between sessions is limited. Under these conditions, skeletal muscle is exposed to increased oxidative stress, inflammatory activation, and structural disruption. These responses may contribute to soreness, transient reductions in force-generating capacity, impaired training quality, and delayed readiness for subsequent exercise [1]. Although these responses are part of normal adaptation, excessive or poorly resolved stress may impair recovery, which has increased interest in nutritional strategies that attenuate exercise-induced biological stress without clearly compromising training adaptation.

Astaxanthin (3,3′-dihydroxy-β,β′-carotene-4,4′-dione) is a lipid-soluble xanthophyll carotenoid that has attracted increasing attention in this context. Owing to its chemical structure, astaxanthin can interact with lipid membranes, scavenge reactive species, and potentially protect cellular structures against peroxidative injury. It has also been linked to inflammatory modulation, membrane stability, and mitochondrial function [2,3,4]. These properties make it a biologically plausible candidate for influencing the response to strenuous exercise, particularly in relation to exercise-induced muscle damage and post-exercise recovery [5,6].

Human trials, however, have produced mixed findings. On the one hand, several randomized controlled trials (RCTs) have suggested that astaxanthin supplementation may attenuate biomarkers of muscle damage, reduce subjective soreness, or improve indices of post-exercise recovery [7]. On the other hand, not all trials have shown favorable effects on inflammatory or oxidative stress markers, and null findings remain common across both trained and recreationally active populations [8,9].

The evidence is even less consistent when exercise performance is considered. In theory, these biological effects could translate into better endurance, delayed fatigue, or improved sport-specific output [10]. Indeed, some studies have reported improvements in cycling time-trial performance, time to exhaustion, or selected sport-specific tasks following supplementation [11]. However, other well-conducted trials have found no clear benefit for VO_2_max, fat oxidation, time-trial performance, or maximal workload [12,13,14]. This inconsistency suggests that any ergogenic effect of astaxanthin is unlikely to be universal and may depend on participant characteristics, supplementation dose and duration, exercise model, and the specific endpoint examined.

A major challenge in this literature is that recovery biomarkers and performance outcomes are often discussed together despite reflecting different levels of response. Biomarkers such as CK, LDH, MDA, and inflammatory mediators are closer to the proposed biological actions of astaxanthin, whereas VO_2_max, time-trial performance, maximal workload, and sport-specific tests are influenced by many additional factors. Astaxanthin may therefore affect recovery without producing a clear improvement in integrated performance outcomes. Distinguishing these domains is essential for a balanced interpretation of the evidence.

To date, although narrative reviews have summarized the antioxidant and general health-related properties of astaxanthin [15], a focused synthesis of randomized controlled trials evaluating both exercise recovery biomarkers and exercise performance remains limited. Clarifying whether the available evidence supports a predominantly recovery-oriented role, a performance-oriented role, or a context-dependent role for astaxanthin is therefore important for both research and practice. Accordingly, this systematic review and meta-analysis aimed to evaluate the effects of astaxanthin supplementation on exercise recovery biomarkers and exercise performance in healthy participants and athletic populations.

## 2. Materials and Methods

### 2.1. Study Design and Reporting Standards

This systematic review and meta-analysis was conducted in accordance with the PRISMA 2020 statement and the Cochrane Handbook for Systematic Reviews of Interventions [16]. The protocol was developed a priori and registered in PROSPERO (CRD420251119762) before study screening and data extraction. Any deviations from the protocol were documented and justified in the Supplementary Materials. The PRISMA 2020 checklist is provided in Supplementary Section S5 [16].

### 2.2. Information Sources and Search Strategy

A comprehensive literature search was conducted in PubMed/MEDLINE, Web of Science Core Collection, Embase, EBSCOhost (SPORTDiscus), the Cochrane Library, and China National Knowledge Infrastructure (CNKI) from database inception to January 2026. Trial registries were also searched where relevant. Forward citation tracking of included trials was performed using Google Scholar, and conference proceedings were screened where available. The search strategy combined controlled vocabulary and free-text terms related to astaxanthin, exercise performance, recovery, and randomized controlled trials. Boolean operators and database-specific filters were applied, without language restrictions where feasible. Reference lists of included trials and relevant reviews were hand-searched. When the source or form of astaxanthin was unclear, supplementary files, manufacturer information, trial registry entries, and author contact were used for clarification. This multi-source search approach was consistent with recent systematic review and network meta-analysis practice in exercise and physical activity research [17]. The full search strategies for all databases are provided in Supplementary Section S1.

### 2.3. Eligibility Criteria

Eligibility was defined using the PICOS framework. We included randomized controlled trials enrolling healthy adolescents and adults (≥16 years), including recreationally active individuals and trained athletes. Clinical populations were excluded unless participants were otherwise described as healthy and the intervention primarily targeted exercise-related outcomes.

Eligible interventions were oral astaxanthin-containing supplements. Single-ingredient formulations were preferred for the primary pooled analyses. Multi-ingredient products or co-interventions were also considered eligible when astaxanthin was a principal active component, when this study was informative for the broader evidence base, or when outcome-level inclusion remained interpretable. These studies were examined cautiously in sensitivity analyses and discussion. Astaxanthin source was classified as natural, synthetic, or unclear based on explicit statements in the manuscript, supplement labels, trial registry entries, or manufacturer documentation. Natural-source formulations were preferred where reported because they improve biological interpretability, but studies were not excluded solely because source details were incompletely reported. When source information remained uncertain after attempts to obtain clarification, this study was retained, flagged as “source unclear,” and examined in sensitivity analyses.

Comparators included placebo, inert control, or no supplementation. Trials with no-supplementation comparators were eligible, but because the absence of placebo may introduce expectation effects, these studies were examined in sensitivity analyses excluding non-placebo-controlled designs.

Eligible studies had to report at least one exercise-related outcome. Performance outcomes included VO_2_max/VO_2_peak, time-trial or time-to-exhaustion tests, maximal or peak power, strength outcomes, sprint metrics, and sport-specific performance tests. Recovery outcomes included biomarkers of muscle damage (e.g., creatine kinase, lactate dehydrogenase), inflammation (e.g., CRP, IL-6, TNF-α), oxidative stress or antioxidant status (e.g., MDA, TAC, SOD, GPx), and subjective outcomes such as delayed-onset muscle soreness or perceived fatigue. Both parallel-group and randomized crossover RCTs were eligible. Observational studies, non-randomized trials, conference abstracts without full text, case reports, editorials, and animal or in vitro studies were excluded.

### 2.4. Study Selection

All records were imported into EndNote 2025 and duplicates were removed. Two reviewers (S.L., W.Y) independently screened titles and abstracts and then assessed the full text of potentially eligible reports against the predefined criteria. Standardized eligibility forms were used, and reviewers were blinded to each other’s decisions where feasible. Disagreements were resolved by discussion, and a third reviewer adjudicated unresolved cases. Inter-reviewer agreement at the title/abstract stage was quantified using Cohen’s kappa and was almost perfect (κ = 0.90) [18]. The study selection process is summarized in a PRISMA 2020 flow diagram [19].

### 2.5. Data Extraction

Two reviewers (S.L., W.Y) independently extracted data using a pre-piloted form. Extracted variables included study characteristics, participant characteristics, intervention details, exercise protocols, and outcome data. Means and standard deviations at baseline and post-intervention were extracted whenever available, and change scores were extracted directly when reported. Data available only in figures were digitized using GetData Graph Digitizer, and standard errors, confidence intervals, and *p* values were converted to standard deviations when necessary.

When change-score standard deviations were missing, they were calculated using an assumed within-study correlation (primary assumption r = 0.5), with sensitivity analyses using r = 0.3 and r = 0.7 [20]. Outcome units were harmonized where feasible; for example, VO_2_ outcomes were converted to mL·kg^−1^·min^−1^ when sufficient information was available. When key information was unavailable, corresponding authors were contacted up to three times over four weeks. Outcomes with unavailable essential data were excluded from quantitative synthesis and summarized narratively. Several eligible recovery-related outcomes were not meta-analyzed because too few studies reported sufficiently comparable measures, timepoints, or units to support quantitative synthesis.

To improve comparability across studies, pre-specified decision rules were applied for outcomes, timepoints, and multiple measures of the same construct. When multiple post-intervention assessments were reported, the timepoint closest to the end of supplementation was selected for the primary analysis. Additional timepoints were summarized narratively or examined in sensitivity analyses where appropriate. When multiple measures reflected the same construct, a pre-specified hierarchy was used; for example, VO_2_max was prioritized over VO_2_peak when maximal criteria were explicitly reported. The full outcome hierarchy is provided in the Supplementary Materials. For multi-arm trials, one independent effect estimate per study per outcome was included in each synthesis. When dose-specific contrasts were not required, intervention arms were combined using Cochrane-recommended methods; when dose-specific contrasts were required, the shared comparator group was split evenly across comparisons. Extracted outcomes were grouped into performance-related and recovery-related domains according to a pre-specified framework. The overall structure of outcome classification was informed by recent evidence syntheses in sports nutrition that evaluated supplement effects within sport- and context-specific performance domains [21,22,23].

### 2.6. Risk of Bias Assessment

Risk of bias was assessed independently by two reviewers (S.L., W.Y) using the Cochrane RoB 2 tool [24]. The following domains were evaluated: randomization process, deviations from intended interventions, missing outcome data, measurement of the outcome, and selection of the reported result. Each domain and the overall judgment were classified as low risk, some concerns, or high risk.The study-level risk-of-bias summary is provided in Supplementary Section S2, Figure S1.

### 2.7. Statistical Analysis

The primary analyses were conducted within a frequentist random-effects meta-analysis framework. For outcomes reported on a common scale, mean differences (MDs) were used; otherwise, standardized mean differences (SMDs) were calculated as Hedges’ g. Change-from-baseline values were prioritized; if unavailable, post-intervention values were used. Effect directions were harmonized so that positive values consistently indicated a favorable effect of astaxanthin, and outcomes for which lower values reflected improvement were reverse-coded before pooling.

The pre-specified primary outcomes were cardiorespiratory fitness (VO_2_max/VO_2_peak), maximal or peak power output, endurance performance (time-trial or time-to-exhaustion, selected according to a pre-specified hierarchy), and creatine kinase (CK). All other outcomes were considered secondary or exploratory. To reduce multiplicity, interpretation focused primarily on pooled estimates for the primary outcomes, whereas secondary outcomes were considered supportive and hypothesis-generating.

Random-effects meta-analyses were performed using restricted maximum likelihood (REML) estimation with the Hartung–Knapp–Sidik–Jonkman adjustment [25].

### 2.8. Subgroup and Sensitivity Analyses

Pre-specified subgroup analyses were conducted, where data permitted, by training status (trained vs. recreationally active), astaxanthin dose (<10 vs. ≥10 mg/day), and intervention duration (<4 vs. ≥4 weeks). These cutoffs were selected a priori because 10 mg/day separated lower-dose protocols (4–8 mg/day) from the moderate-to-high-dose protocols most commonly represented in the included trials, whereas 4 weeks distinguished acute or short-term supplementation from longer repeated-dosing interventions. Subgroup analyses were performed only when each subgroup contained an adequate number of independent estimates. Meta-regression was planned only when at least 10 independent study estimates were available.

Sensitivity analyses included leave-one-out analyses; exclusion of studies at high risk of bias; exclusion of crossover trials; re-analysis using alternative assumed correlations (r = 0.3 and r = 0.7); exclusion of non-placebo-controlled studies; exclusion of studies with unclear astaxanthin source; and restriction to studies reporting change scores where feasible.

### 2.9. Publication Bias

Publication bias and small-study effects were explored for all analyzable outcomes. Funnel plots were visually inspected, and Egger’s regression test was additionally conducted in the complementary frequentist analyses as exploratory assessments [26].

### 2.10. Certainty of Evidence

The certainty of evidence for the pre-specified primary outcomes was assessed using the GRADE approach across the domains of risk of bias, inconsistency, indirectness, imprecision, and publication bias [27]. Assessments were conducted at the outcome level and summarized in the Summary of Findings table (Table 2). Secondary outcomes were treated as supportive evidence.

### 2.11. Software

All analyses were performed using R (version 4.5.0). Meta-analyses were conducted using a frequentist random-effects framework with restricted maximum likelihood estimation and the Hartung–Knapp–Sidik–Jonkman adjustment. Additional analyses and visualizations were generated using relevant R packages.

## 3. Results

### 3.1. Study Selection

**Figure 1:**
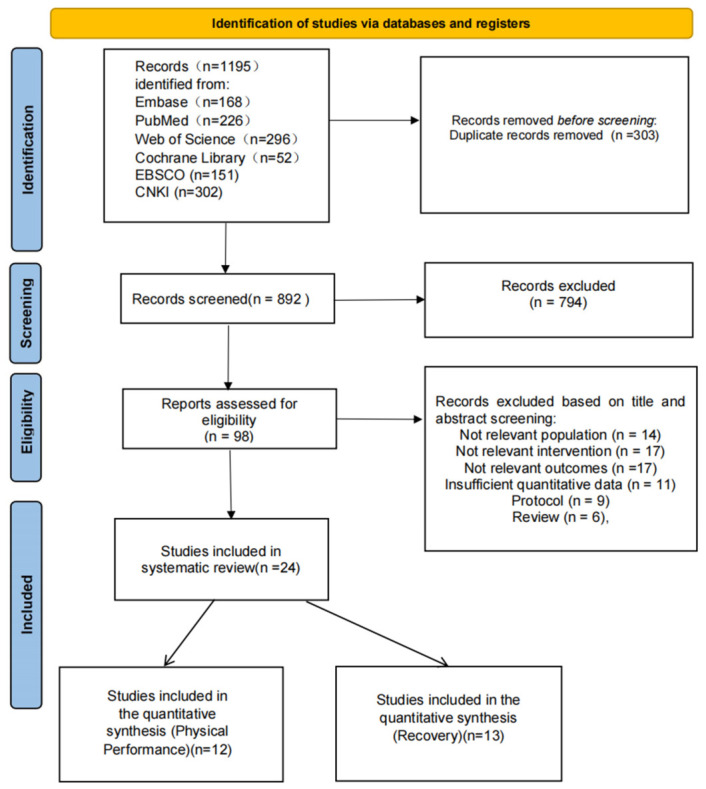
Screening flowchart.

According to the PRISMA flow diagram (Figure 1), a total of 1195 records were identified from electronic databases, including Embase (n = 168), PubMed (n = 226), Web of Science (n = 296), Cochrane Library (n = 52), EBSCO (n = 151), and CNKI (n = 302). After removal of 303 duplicate records, 892 records remained for title and abstract screening, of which 794 were excluded. A total of 98 reports were assessed for full-text eligibility, and 74 were excluded for the following reasons: not-relevant population (n = 14), not-relevant intervention (n = 17), not-relevant outcomes (n = 17), insufficient quantitative data (n = 11), protocol (n = 9), and review article (n = 6). Ultimately, 24 randomized controlled trials were included in the systematic review. Of these, 12 studies contributed to the quantitative synthesis of physical performance outcomes and 13 studies contributed to the quantitative synthesis of recovery-related outcomes, as shown in Figure 1.

### 3.2. Study Characteristics

The 24 included randomized controlled trials enrolled a broad range of physically active populations, including trained cyclists, soccer players, resistance-trained men, recreationally active adults, runners, firefighters, older adults, and sport-specific athletes. Astaxanthin doses ranged from 4 to 28 mg/day and intervention durations from 4 days to 16 weeks. All astaxanthin formulations were provided as capsules. Exercise models were heterogeneous and included cycling time trials, graded exercise tests, exhaustive aerobic protocols, eccentric exercise-induced muscle damage models, resistance exercise, and sport-specific tests. Reported outcomes covered both performance indices and recovery-related biomarkers. Studies involving multi-ingredient formulations or co-interventions were retained transparently in the review, and their contribution to pooled analyses was judged outcome by outcome according to data structure and interpretability. The main characteristics of the included studies are summarized in Table 1, and additional study-level context is provided in the Supplementary Materials.

### 3.3. Risk of Bias

**Figure 2:**
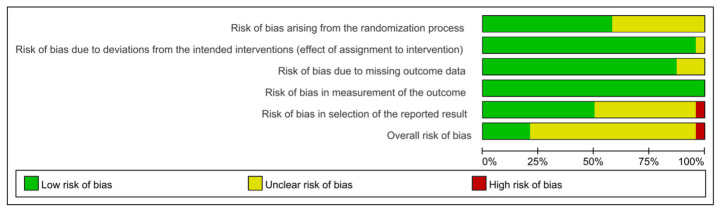
Risk-of-bias assessment.

The overall methodological quality of the included studies was mixed (Figure 2). Most studies were judged as low risk of bias in the domains of deviations from intended interventions, missing outcome data, and measurement of the outcome. By contrast, the randomization process and selection of the reported result were the two domains in which concerns were most frequently identified. At the overall level, a substantial proportion of studies were judged as having some concerns, while only a small number were classified as high risk of bias. This pattern suggests that, although the included evidence was largely derived from randomized and generally well-conducted trials, uncertainty remained mainly in relation to inadequate reporting of randomization procedures and selective outcome reporting. These issues were taken into account in the subsequent GRADE assessment and in the interpretation of pooled results. A more detailed visual summary is provided in Supplementary Section S2, Figure S1.

### 3.4. Recovery-Related Outcomes

Recovery-related outcomes focused on post-exercise biomarkers, including CK, IL-6, LDH, and MDA.

#### 3.4.1. Creatine Kinase (CK)

**Figure 3:**
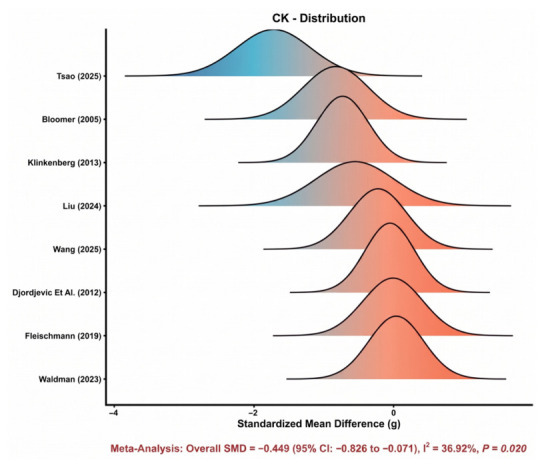
Distribution of study-specific effect sizes for CK [8,9,12,29,33,44,45,47].

Eight comparisons were included in the meta-analysis of CK. As shown in Figure 3, most study-specific effect estimates were distributed on the beneficial side of the null, although the magnitude of the effect varied across studies. The pooled analysis showed that astaxanthin supplementation significantly reduced CK compared with control (SMD = −0.45, 95% CI: −0.83 to −0.07), indicating a favorable effect on exercise-induced muscle damage. Between-study heterogeneity was moderate (*I*^2^ = 36.9%), suggesting that the direction of effect was relatively consistent despite some variation in effect size. Overall, Figure 3 supports a modest but directionally coherent recovery benefit for CK.

#### 3.4.2. Interleukin-6 (IL-6)

Three comparisons contributed to the IL-6 meta-analysis. The pooled effect showed no statistically significant influence of astaxanthin supplementation on IL-6 (SMD = −0.23, 95% CI: −0.65 to 0.20), and statistical heterogeneity was absent (*I*^2^ = 0.0%). Supplementary Section S3, Figures S2A and S3A provide the corresponding distribution and precision plots.

#### 3.4.3. Lactate Dehydrogenase (LDH)

**Figure 4:**
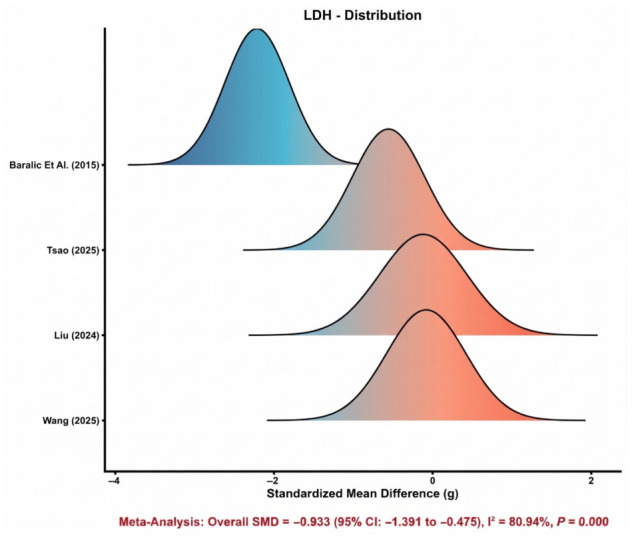
Distribution of study-specific effect sizes for LDH [31,44,45,47].

Four comparisons were synthesized for LDH. Figure 4 shows that the study-specific effect estimates were more widely dispersed than those observed for CK, indicating greater variability among trials. Nevertheless, the pooled estimate favored astaxanthin supplementation and demonstrated a significant reduction in LDH compared with control (SMD = −0.93, 95% CI: −1.39 to −0.48). This suggests a potentially meaningful protective effect on exercise-related tissue damage. However, heterogeneity was substantial (*I*^2^ = 80.9%), indicating that the magnitude of benefit differed considerably across studies. Therefore, while the direction of effect was favorable overall, the strength and generalizability of the LDH finding should be interpreted with caution.

#### 3.4.4. Malondialdehyde (MDA)

Six comparisons were included for MDA. The pooled result did not reach statistical significance (SMD = −0.22, 95% CI: −0.59 to 0.14), although the direction of effect favored astaxanthin supplementation. Heterogeneity was low to moderate (*I*^2^ = 12.0%), suggesting relatively limited between-study variability. The corresponding distribution and precision plots are provided in Supplementary Section S3, Figure S2B, and Supplementary Section S4, Figure S3B, respectively.

#### 3.4.5. Summary of Recovery-Related Outcomes

Overall, the recovery-related outcomes more clearly supported attenuation of exercise-induced muscle damage than modulation of inflammatory or oxidative stress markers. CK provided the most consistent favorable signal. LDH also favored astaxanthin, but this finding should be interpreted cautiously because of substantial heterogeneity and sensitivity to individual studies. IL-6 and MDA remained non-significant.

### 3.5. Exercise Performance Outcomes

#### 3.5.1. VO_2_max

**Figure 5:**
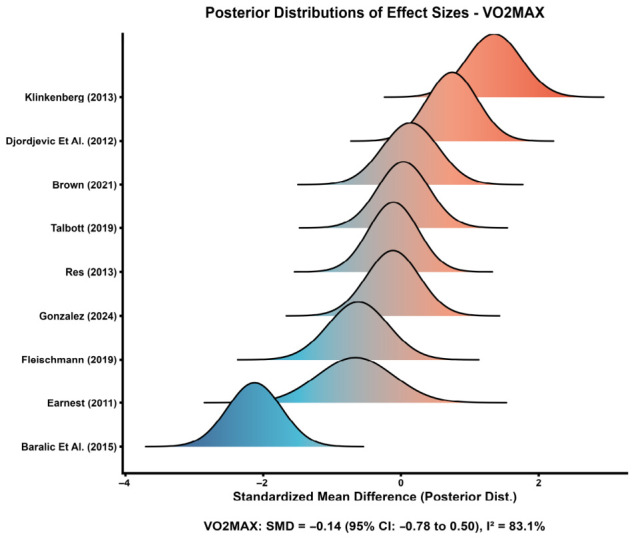
Distributions of study-specific effect sizes for VO_2_max [12,14,29,31,33,34,36,43,47].

Nine comparisons contributed to the pooled analysis for VO_2_max. As shown in Figure 5, the study-specific effect estimates were widely distributed on both sides of the null, visually reflecting marked between-study heterogeneity. The pooled effect indicated no significant improvement in VO_2_max with astaxanthin supplementation compared with control (SMD = −0.14, 95% CI: −0.78 to 0.50), and heterogeneity was considerable (*I*^2^ = 83.1%). These findings suggest that any effect of astaxanthin on cardiorespiratory fitness is likely to be small, inconsistent, or dependent on specific study conditions. An additional precision plot for VO_2_max is provided in Supplementary Section S4, Figure S3E.

#### 3.5.2. Time-Trial Performance

Three comparisons were available for time-trial performance. The pooled estimate showed no significant effect of astaxanthin supplementation on time-trial performance (SMD = −0.17, 95% CI: −0.76 to 0.42), with moderate heterogeneity (*I*^2^ = 43.7%). The corresponding distribution and precision plots are provided in Supplementary Section S3, Figure S2C, and Supplementary Section S4, Figure S3C, respectively.

#### 3.5.3. Maximal Workload/Power-Related Outcomes

Five comparisons contributed to the pooled analysis for maximal workload/power-related outcomes. This category included closely related endpoints reported across the original trials, such as WRmax, maximal workload, peak workload, or peak power. No statistically significant between-group difference was observed (SMD = −0.11, 95% CI: −0.53 to 0.31), with low heterogeneity (*I*^2^ = 23.4%).The corresponding distribution and precision plots are provided in Supplementary Section S3, Figure S2D, and Supplementary Section S4, Figure S3D, respectively.

#### 3.5.4. Summary of Performance Outcomes

Overall, the pooled evidence did not support a significant ergogenic effect of astaxanthin on the main performance outcomes. Although some individual trials reported benefits under specific conditions, the pooled results for VO_2_max, time-trial performance, and WRmax were all non-significant. Any direct performance-enhancing effect, if present, is therefore likely to be small and context dependent.

### 3.6. Subgroup Analyses

Because only a limited number of studies contributed to each pooled outcome, formal meta-regression was not performed. Pre-specified subgroup analyses were instead conducted for VO_2_max and CK according to exercise type, intervention duration, and dose.

**Figure 6:**
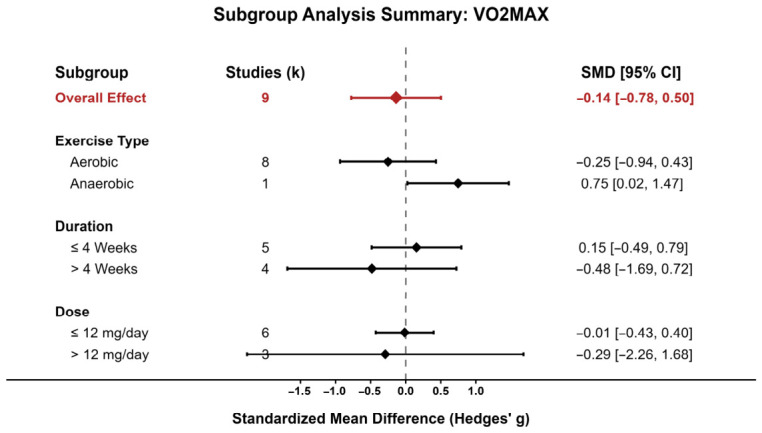
VO_2_max subgroup analysis.

For VO_2_max, subgroup results are summarized in Figure 6. No consistent subgroup pattern indicating a clear performance-enhancing effect was observed. The overall pooled effect remained close to the null, and most subgroup confidence intervals crossed zero. Although the anaerobic subgroup showed a positive point estimate, this result was based on only one study and should therefore be interpreted cautiously. Likewise, no clear modifying effect of intervention duration or dose was evident, as both shorter versus longer interventions and lower versus higher doses produced overlapping confidence intervals.

**Figure 7:**
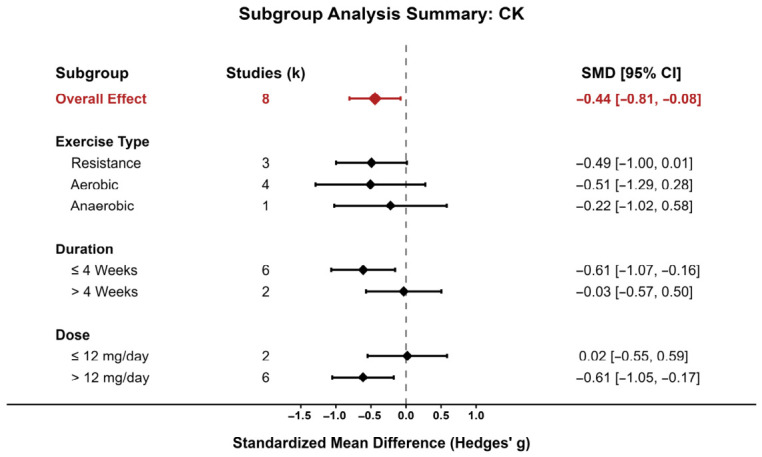
CK subgroup analysis.

For CK, subgroup results are shown in Figure 7. The overall subgroup pattern suggested that the reduction in CK was more evident in the higher-dose subgroup (≥10 mg/day) and shorter interventions (<4 weeks). In contrast, studies with lower doses or longer durations showed effect estimates closer to the null. Across exercise types, the direction of effect generally favored astaxanthin, although confidence intervals remained wide in several subgroups because of the limited number of studies.

### 3.7. Exploratory Precision Plots

**Figure 8:**
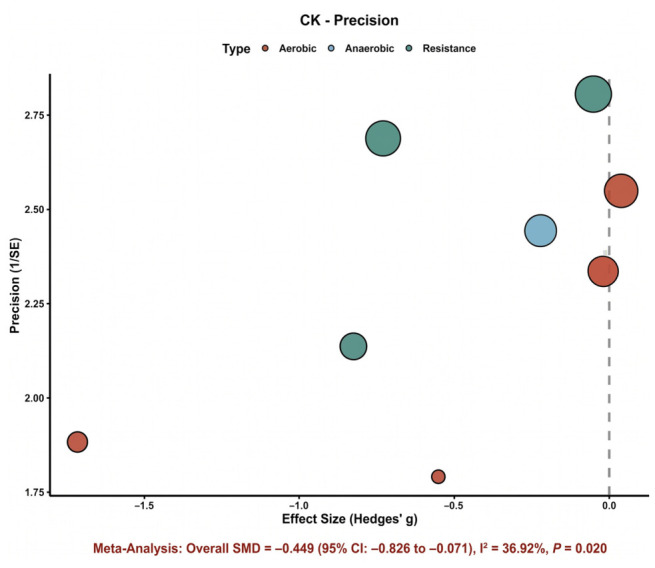
CK precision plot. Each bubble represents one study-specific comparison. Bubble size is proportional to the precision of the effect estimate, with larger bubbles indicating higher precision and smaller standard errors. The dashed vertical line represents the null effect.

**Figure 9:**
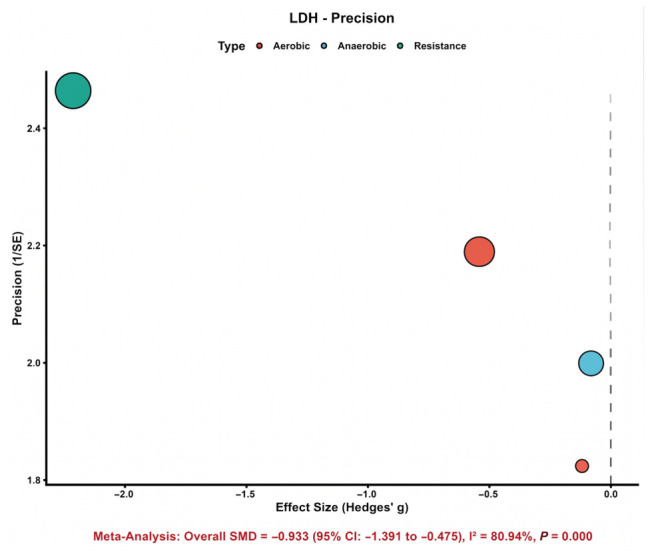
LDH precision plot. Each bubble represents one study-specific comparison. Bubble size is proportional to the precision of the effect estimate, with larger bubbles indicating higher preci-sion and smaller standard errors. The dashed vertical line represents the null effect.

Exploratory precision plots provided additional support for these interpretations. In the CK bubble plot (Figure 8), most studies clustered on the beneficial side of the null with moderate-to-high precision, supporting the relative stability of the pooled CK effect. By contrast, the VO_2_max bubble plot (Figure 9) showed a wider spread of study estimates on both sides of the null, consistent with the substantial heterogeneity observed in the main analysis. Overall, these graphical patterns support the conclusion that the evidence for CK is more coherent than that for VO_2_max. Supplementary Figure S3A–E extend these exploratory plots to additional outcomes.Additional exploratory precision plots for IL-6, MDA, time-trial performance, maximal workload/power-related outcomes, and VO_2_max are provided in Supplementary Section S4, Figures S3A–S3E.

### 3.8. Sensitivity Analyses

Leave-one-out sensitivity analyses were performed for outcomes showing substantial heterogeneity. For LDH (*I*^2^ = 80.9%), omission of Baralic et al. (2015) [31] reduced heterogeneity markedly to 0%, and the pooled effect became non-significant, suggesting that this study was the principal source of heterogeneity and had a strong influence on the overall estimate. For TT (*I*^2^ = 43.7%), exclusion of Liu et al. (2018) [32] reduced heterogeneity to 0%, although the pooled effect remained non-significant, indicating that this study mainly contributed to between-study inconsistency rather than altering the overall conclusion. For VO_2_max (*I*^2^ = 83.1%), omission of Baralic et al. (2015) [31] reduced heterogeneity from 83.1% to 61.0%, but substantial heterogeneity remained, suggesting that the inconsistency in VO_2_max was likely multi-source rather than attributable to a single study.

### 3.9. Publication Bias

**Figure 10:**
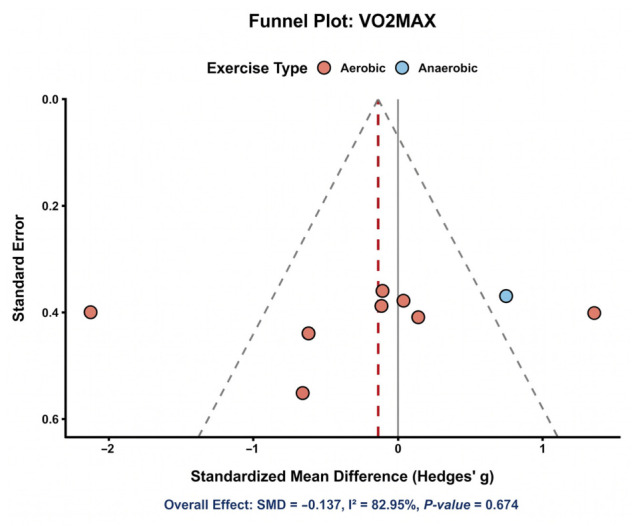
VO_2_max funnel plot. Each point represents one study-specific comparison. The solid vertical line represents the null effect (SMD = 0), the vertical dashed line represents the pooled overall effect estimate, and the diagonal dashed lines indicate the approximate 95% pseudo-confidence limits around the pooled effect. Points are colored according to exercise type.

**Figure 11:**
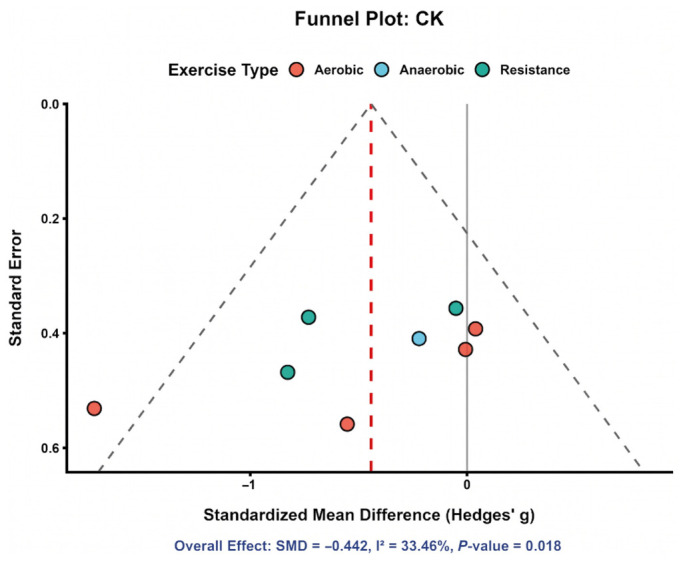
CK funnel plot. Each point represents one study-specific comparison. The solid vertical line represents the null effect (SMD = 0), the vertical dashed line represents the pooled overall effect estimate, and the diagonal dashed lines indicate the approximate 95% pseudo-confidence limits around the pooled effect. Points are colored according to exercise type.

Formal assessment of publication bias was constrained by the limited number of studies available for most outcomes. Nevertheless, funnel plots were generated for selected outcomes, including VO_2_max and CK, for exploratory visual inspection. The VO_2_max funnel plot (Figure 10) did not show clear asymmetry, although interpretation was limited by substantial heterogeneity. Similarly, the CK funnel plot (Figure 11) did not reveal obvious asymmetry, but conclusions remained tentative because of the relatively small number of included comparisons. Publication bias therefore could neither be confidently confirmed nor excluded and was interpreted cautiously in conjunction with the GRADE assessment.

### 3.10. Certainty of Evidence

Using GRADE, the certainty of evidence ranged from moderate to very low across outcomes (Table 2). CK provided the most consistent recovery-related signal and was rated as moderate-certainty evidence. LDH was rated as low certainty because of substantial heterogeneity. MDA and IL-6 were rated as very low certainty because of small study numbers, serious imprecision, and the possibility of small-study effects. For performance outcomes, VO_2_max, time-trial performance, and WRmax were all rated as low certainty.

**Table 2:** GRADE summary of findings.

GRADE Summary of Findings											
*Astaxanthin Supplementation Versus Placebo/Control in Healthy Adults and Athletes*											
Outcomes	Certainty Assessment							No. of Participants		Effect Size (Hedges’ g, 95% CI)	Certainty
	No. of Studies	Study Design	Risk of Bias	Inconsistency	Indirectness	Imprecision	Other Considerations	Astaxanthin	Control		
**Exercise Recovery**											
**CK**	8	Randomized trials	Not serious	Not serious	Not serious	Serious	None	113	85	**−0.45 (−0.83 to −0.07)**	**⨁⨁⨁◯**** Moderate**
**IL-6**	3	Randomized trials	Serious	Not serious	Not serious	Very serious	Publication bias could not be ruled out	55	39	**−0.23 (−0.65 to 0.20)**	**⨁◯◯◯**** Very low**
**LDH**	4	Randomized trials	Serious	Serious	Not serious	Not serious	None	44	47	**−0.93 (−1.39 to −0.48)**	**⨁⨁◯◯**** Low**
**MDA**	6	Randomized trials	Serious	Not serious	Not serious	Very serious	Publication bias could not be ruled out	71	71	**−0.22 (−0.59 to 0.14)**	**⨁◯◯◯**** Very low**
**Exercise Performance**											
**VO_2_max**	9	Randomized trials	Serious	Serious	Not serious	Not serious	None	128	121	−0.14 (−0.78 to 0.50)	**⨁⨁◯◯**** Low**
**Time-trial performance**	3	Randomized trials	Serious	Not serious	Not serious	Serious	None	42	45	**−0.17 (−0.76 to 0.42)**	**⨁⨁◯◯**** Low**
**WRmax**	5	Randomized trials	Serious	Not serious	Not serious	Serious	None	61	57	**−0.11 (−0.53 to 0.31)**	**⨁⨁◯◯**** Low**

Note: In the GRADE certainty ratings, ⨁ indicates one level of certainty and ◯ indicates a downgraded or empty level.

## 4. Discussion

### 4.1. Principal Findings

This systematic review and meta-analysis suggests that astaxanthin may be more relevant to exercise recovery than to direct enhancement of exercise performance. In the pooled analyses, CK provided the most consistent favorable signal, whereas the apparent benefit for LDH should be interpreted cautiously because of substantial heterogeneity and sensitivity to individual studies. By contrast, no statistically robust pooled effects were identified for VO_2_max, time-trial performance, or WRmax. Taken together, the current evidence does not support astaxanthin as a universally effective ergogenic aid, but it does support a possible recovery-supportive role in some exercise settings.

A second key point is that the certainty of evidence was not uniform across outcomes. CK emerged as the most credible positive recovery signal, whereas LDH was favorable but less secure because of greater heterogeneity. By contrast, most performance outcomes remained low-certainty and inconsistent. This pattern suggests that the practical value of astaxanthin, if any, is more likely to lie in supporting recovery from exercise stress than in producing large and reliable improvements in aerobic fitness or competitive output across all settings.

### 4.2. Recovery-Related Outcomes: Why CK and LDH Are the Clearest Signals

Among all outcomes, CK is the most convincing positive finding in the present review. CK is widely used as an indirect indicator of exercise-induced muscle membrane disruption, and although it is an imperfect marker, it remains highly relevant in studies of strenuous exercise and recovery [48,49]. In the current synthesis, the pooled reduction in CK was modest but directionally consistent, and the certainty of evidence was higher than for most other endpoints. This makes CK the most stable indicator that astaxanthin may blunt at least part of the muscle-damage response to intense exercise.

Physiologically, CK primarily reflects leakage of a cytosolic muscle enzyme after disruption of sarcolemmal integrity, particularly after eccentric, exhaustive, or unaccustomed exercise. A smaller CK response may therefore indicate attenuated membrane permeability, less secondary muscle-cell disruption, or a faster return toward cellular homeostasis, although CK remains influenced by training status, exercise mode, and sampling time.

Trial-level findings were broadly consistent with this interpretation. Several studies reported lower CK or related muscle-damage markers after astaxanthin supplementation during heavy training or exhaustive exercise, although the limited number of trials and variation in dose, duration, and exercise model prevent firm conclusions about dose-response effects [11,29,31].

LDH showed an even larger pooled effect than CK, but it should be interpreted more cautiously. The magnitude of the pooled reduction was favorable, yet between-study heterogeneity was substantial. This indicates that the magnitude of benefit varied considerably across studies. Therefore, although the overall direction favored astaxanthin, the precision, generalizability, and robustness of the LDH finding remain less certain than those for CK.

From a practical perspective, lower CK and LDH may indicate a reduced biochemical burden of exercise-induced muscle disruption, which could be relevant in sport and training contexts involving repeated high-intensity sessions, congested competition schedules, heavy eccentric loading, or limited recovery intervals. However, these biomarkers should not be interpreted as direct surrogates for functional recovery, soreness resolution, or improved subsequent exercise performance.

LDH is also released when cellular membranes are stressed or damaged, but it is less muscle-specific than CK because it is present in several tissues and is affected by tissue source, exercise modality, and post-exercise sampling window. This broader biological origin may help explain why the pooled LDH effect appeared favorable but was less stable and more heterogeneous than the CK finding.

Mechanistically, the CK and LDH findings fit what is already known about astaxanthin. Because astaxanthin can localize within lipid structures and may help reduce oxidative injury to cell membranes, it is reasonable to expect that leakage of intracellular enzymes after strenuous exercise could be attenuated [2,3,49]. That interpretation does not require claiming that astaxanthin prevents all exercise-induced damage, nor that it improves every recovery dimension. It only requires a more modest conclusion: in some exercise settings, astaxanthin may reduce the biochemical burden of muscle disruption and thereby improve recovery conditions.

### 4.3. Other Recovery Biomarkers: Supportive but Weaker Evidence

Evidence for oxidative stress and inflammatory markers was weaker than that for CK and LDH. MDA and IL-6 showed no stable pooled signal, and the certainty of evidence was very low. This does not necessarily indicate a complete absence of effect; rather, these outcomes were more difficult to synthesize because studies differed in exercise model, sampling window, dose, and participant characteristics.

For oxidative and inflammatory recovery markers, MDA and IL-6 capture different physiological processes. MDA is a downstream product of lipid peroxidation and is commonly interpreted as an index of oxidative injury to polyunsaturated fatty acids within cell membranes. IL-6 is a pleiotropic cytokine and contraction-related myokine that can rise transiently with muscle contraction, glycogen depletion, and systemic inflammatory activation; therefore, its interpretation depends strongly on exercise intensity, nutritional state, and timing of blood sampling.

Even so, some individual trials still provide useful context. Tsao et al. [11] observed lower MDA after exhaustive cycling, and Wang et al. [45] found that medium and high doses improved MDA, SOD, and TNF-α responses after exhaustive exercise, although the best functional recovery was again seen at 12 mg/day rather than 24 mg/day. Wu et al. [35] reported that 12 mg/day reduced post-exercise blood lactate and preserved antioxidant capacity after acute high-intensity exercise, while Wang et al. (2021) [38], using metabolomics, suggested faster post-exercise metabolic recovery with astaxanthin supplementation. These studies do not overturn the low-certainty pooled evidence, but they do help explain why recovery-related effects remain biologically credible even when not every biomarker is consistently significant in meta-analysis.

### 4.4. Exercise Performance: Pooled Null Findings, but Literature That Is Not Completely Negative

For exercise performance, the most defensible interpretation is that the pooled evidence remains limited and inconsistent rather than uniformly negative. The meta-analysis did not support clear benefits for VO_2_max, time-trial performance, or WRmax, and certainty was low across these outcomes. Current evidence therefore does not justify presenting astaxanthin as a reliable performance-enhancing supplement across populations or exercise modes, even though isolated trials have reported benefits under specific conditions.

That said, the trial-level literature is not entirely null. Some individual trials reported improvements in cycling time-trial performance, time to exhaustion, or sport-specific kicking performance [11,28,36,46], suggesting that astaxanthin may have context-specific effects under selected exercise models.

However, several well-controlled trials reported no meaningful improvements in fat oxidation, VO_2_-related outcomes, time-trial performance, or other performance-related indices [12,13,14,33]. These null findings are consistent with the pooled results and indicate that performance benefits, if present, are unlikely to be universal.

Studies on older adults or lower-fitness populations suggest that astaxanthin-based formulations combined with exercise training may improve walking capacity, muscle function, or substrate metabolism [32,37,40]. However, because these studies often involved special populations or multi-ingredient formulations, they should be interpreted as supportive qualitative evidence rather than direct evidence for single-ingredient astaxanthin effects.

### 4.5. Why the Exercise-Performance Literature Is So Inconsistent

Several factors likely contribute to the inconsistency of the performance literature, including heterogeneity in participant training status, major differences in exercise models, and substantial variation in dose and duration. These differences make direct comparison difficult and may obscure small context-specific effects. A dose-related pattern is possible, but current evidence remains insufficient to define an optimal dose.

### 4.6. Implications, Strengths, and Limitations

From a practical perspective, the present findings do not support recommending astaxanthin as a universal supplement for improving aerobic fitness or maximal performance. A more defensible interpretation is that its value may lie primarily in settings where recovery is critical, such as repeated competition, dense training schedules, heavy eccentric loading, or exercise associated with substantial muscle damage and oxidative stress.

This review also has clear strengths. It focused on randomized controlled trials, separated recovery biomarkers from exercise performance, and combined quantitative synthesis with broader qualitative interpretation. Limitations include the modest number of studies per pooled outcome, substantial methodological heterogeneity, inconsistent biomarker sampling windows, inclusion of both adolescent and adult athletic populations, and the fact that some multi-ingredient or co-intervention studies were more informative for narrative context than for quantitative pooling. An additional limitation is the high proportion of male participants across the included trials, which may restrict generalizability to female athletes or active women given sex-specific differences in substrate metabolism, muscle fiber composition, hormonal milieu, and recovery responses. These limitations warrant caution, but they do not negate the overall pattern that recovery-related evidence is more favorable than performance-related evidence.

## 5. Conclusions

In conclusion, current evidence suggests that astaxanthin supplementation may be more relevant to exercise recovery than to the consistent enhancement of exercise performance. CK provided the most consistent favorable recovery-related signal, whereas the apparent benefit for LDH should be interpreted cautiously. By contrast, pooled evidence for VO_2_max, time-trial performance, and maximal workload remained inconclusive and of limited certainty.

At the same time, the broader literature suggests that performance-related effects cannot be dismissed entirely. Individual trials have reported improvements in cycling time-trial performance, time to exhaustion, walking capacity, and sport-specific tasks under certain conditions, whereas other well-controlled studies found no meaningful effect on aerobic capacity, substrate utilization, or exercise output. Any ergogenic benefit of astaxanthin is therefore likely to be context dependent.

Astaxanthin should not currently be presented as a universal ergogenic aid. A more balanced interpretation is that it may serve as a recovery-supportive nutritional strategy, particularly in settings characterized by high physiological stress or repeated strenuous exercise. Future randomized controlled trials should use larger samples, standardized dosing regimens, consistent post-exercise sampling windows, and clearly separated recovery and performance endpoints.

## Figures and Tables

**Table 1:** Characteristics of the included studies.

Author (Year)	Country/Region	Population	Sample Size (Intervention/Control)	Age	Gender (% Male)	Exercise Type	Intervention (Dose/Duration)	Physical Performance (e.g., TTE, VO_2_max)	Recovery (e.g., CK, LDH, CRP, IL-6, RPE, DOMS)	Result
Bloomer (2005) [9]	USA	Resistance-trained healthy adults	10/10	25.1 ± 1.6	100.0%	Eccentric knee extensions (10 × 10 85% eccentric 1-RM)	Astaxanthin 4 mg/day; 3 weeks pre + 4 days post	1-RM concentric strength (NS), MIF (NS), MDF↓ (*p* < 0.05)	CK; muscle soreness (VAS) (NS)	No benefit on soreness, CK, 1-RM, or MIF; MDF lower vs. placebo at 10–72 h
Earnest (2011) [28]	USA	Competitive male cyclists	7/7	28.0 ± 6.0	100.0%	2 h steady ride (5% below 4 mmol/L lactate threshold) + 20 km TT	Astaxanthin 4 mg/day for 28 days	20 km TT time ↓1; avg. power ↑	Metabolic markers (lactate, glucose, NEFA, glycerol): no significant change	Significant improvement in TT time and power vs. placebo; no substrate metabolism effect
Djordjevic (2012) [29]	Serbia	Elite young male soccer players	18/14	17.9 ± 0.7	100%	In-season soccer training; 2 h soccer-specific exercise bout at day 90	Astaxanthin 4 mg/day for 90 days, oral capsules, double-blind	VO2max measured at baseline for characterization only; no longitudinal performance outcomes reported	TBARS/MDA, AOPP, O_2_•^−^, TAS, SH groups, SOD activity, serum CK and AST measured at baseline, after 90 days supplementation, and pre-/post-2 h soccer exercise at day 90	Astaxanthin attenuated exercise-induced increase in O_2_•^−^ and decrease in TAS, did not change TBARS or AOPP, and reduced post-exercise serum CK and AST compared with placebo
Res (2013) [30]	Netherlands	Well-trained male cyclists/triathletes	16/15	25.0 ± 1.0	100.0%	60 min submax cycling 50% Wmax + 1h TT	Astaxanthin 20 mg/day for 4 weeks	Not significant in TT time (≈59→59 min) or mean power (238→244 W); VO_2_peak unchanged	MDA, TEAC, FFA, glucose, lactate, insulin: no significant changes	Astaxanthin raised plasma level but did not alter metabolism, antioxidant status, or performance
Klinkenberg (2013) [12]	The Netherlands	Well-trained male cyclists	16/15	25.0 ± 5.0	100.0%	60 min steady-state 50% Wmax + 1 h time-trial	Astaxanthin 20 mg/day for 4 weeks	VO_2_max, Wmax: no change	cTnT ↑ after cycling in both groups; ΔcTnT AUC NS (*p* = 0.24); CK, hsCRP, TEAC, MDA unchanged	Plasma astaxanthin ↑↑; no effect on antioxidant capacity, cTnT, CK, hsCRP, or performance
Baralic et al. (2015) [31]	Serbia	Trained young male soccer players	21/19	17.7 ± 0.2	100%	Regular competitive-season soccer training program (strength, resistance, cardio, flexibility, proprioception) over 90 days; no separate laboratory exercise test at follow-up	Astaxanthin 4 mg/day, one capsule with a meal for 90 days, randomized double-blind	VO_2_max measured once at baseline for characterization; no post-supplement performance outcomes reported (physical performance endpoints: not reported)	Salivary sIgA concentration and secretion rate; plasma TAS, TOS, PAB; serum AST, ALT, CK, LDH, UA, creatinine, hs-CRP; hematological profile including leukocyte and neutrophil counts, lipids (CHOL, HDL-C, LDL-C, TG)	Astaxanthin supplementation for 90 days increased resting sIgA concentration and secretion rate, decreased PAB, and, together with regular training, reduced CK, LDH, and AST more clearly than placebo
Liu (2018) [32]	USA	Healthy elderly	23/19	71.0 ± 4.0	47.6%	Incline treadmill interval walking (3×/week, 3 months)	Astaxanthin 12 mg + Tocotrienol 10 mg + Zinc 6 mg/day for 4 months	6 min walk ↑ 8% both groups; MVC ↑14.4% (*p* < 0.02); CSA ↑2.7% (*p* < 0.01); MVC/CSA ↑11.6% (*p* = 0.053)	No blood biomarkers; focus on muscle adaptation	AX group improved muscle strength, CSA, and mobility; placebo group only endurance gains
Fleischmann (2019) [33]	Israel	Healthy young men	12/10	23.1 ± 3.5	100.0%	VO_2_max test + 2 h heat-tolerance walk	Astaxanthin 12 mg/day for 30 days	VO_2_max unchanged; recovery VO_2_ ↓ (–2.02% vs. +0.83%, *p* = 0.001); post-exercise lactate ↓ (9.4 vs. 13.0 mmol/L, *p* < 0.02)	CK, CRP, HSP72 unchanged; HR <120 bpm; RPE mild; sweat rate NS	Astaxanthin improved aerobic recovery but had no effect on heat tolerance or biochemical stress markers
Talbott (2019) [34]	USA	Healthy adults	14/14	42.0 ± 8.0	50.0%	Treadmill VO_2_max test (AeT/AT thresholds)	Astaxanthin 12 mg/day for 8 weeks	VO_2_max unchanged; HR ↓ ~10% at AeT/AT (*p* < 0.05)	Global Mood +11% (*p* < 0.05); Depression –57% (*p* < 0.05); fatigue –36% (*p* < 0.05)	Astaxanthin improved submax HR (cardiotonic effect) and mood balance (heart–brain axis benefit)
Wu (2019) [35]	China	Healthy young men	8/8	19.1 ± 2.5	100.0%	3 × 30 s high-intensity cycling (0.075 kg/kg)	Astaxanthin 12 mg/day for 4 weeks	NP	Antioxidant capacity ↓ post-ex (*p* < 0.05), but higher in AST group (*p* < 0.05); LA ↑ both (*p* < 0.01), lower in AST group (*p* < 0.05); UA ↓ slightly (*p* < 0.05)	Astaxanthin reduced exercise-induced lactate accumulation and enhanced antioxidant defense
Brown (2021) [36]	UK	Recreationally trained male cyclists	12 (crossover)	27.5 ± 5.7	100.0%	40 km cycling time trial	Astaxanthin 12 mg/day × 7 days	40 km TT time ↓1.2% (*p* = 0.029); mean power ↑2.8% (*p* = 0.047)	FATox ↑ (+0.09 g·min^−1^, *p* = 0.044); RER ↓ (*p* = 0.024); lactate, glucose NS	Astaxanthin improved cycling TT performance and enhanced fat oxidation in late exercise stage
Liu (2021) [37]	USA	Healthy older adults	22/18	65–82	42.5%	Treadmill interval aerobic training (12 wks)	Astaxanthin 12 mg/day for 3 months	TA endurance ↑ (+34%, *p* < 0.05); GXT time ↑ (+4 min, *p* < 0.05)	FATox ↑ (+0.76 vs. +0.23 g, *p* < 0.05); CHOox ↓ (*p* < 0.05 in males); RER ↓ (–0.03, *p* < 0.05); efficiency ↑ (males only, *p* < 0.05)	Astaxanthin improved fat oxidation and exercise efficiency, with stronger effects in males
Wang (2021) [38]	China	Healthy male PE students	8/8	20.3 ± 2.4	100.0%	3 × 30 s maximal cycling (0.075 kg/kg)	Astaxanthin 12 mg/day × 4 weeks	No direct performance data	↓β-hydroxybutyrate, ↓glycerol, ↑creatine (1 h post-exercise); ↑methionine, ↓glycerol (1 d)	Astaxanthin accelerated metabolic recovery via improved creatine, amino acid, and lipid metabolism after high-intensity exercise
Guo (2021) [39]	China	Healthy male physical education students (trained, 18–20 y)	8/8	19.0 ± 0.5 (approximate)	100%	Three 30 s bouts of high-intensity cycling at 0.075 kg/kg with 3 min rest	Astaxanthin 12 mg/day, orally, once daily for 28 days	Not reported	Antioxidant capacity index; blood lactate	Astaxanthin increased antioxidant capacity and reduced blood lactate at rest and post-exercise; metabolomics indicated altered amino acid and lipid metabolism pathways
Nakanishi (2022) [40]	Japan	Elderly nursing-home residents	11/13	67–94	NP	6 min walk test	Astaxanthin 24 mg/day × 16 weeks	↑ 6-MWD (*p* < 0.05); no change in strength or mass	lactate (122→90%, *p* < 0.05)	Astaxanthin improved walking endurance and oxidative stress without affecting muscle strength
McAllister (2022) [13]	USA	Active young men	14 (cross-over)	23 ± 2	100%	Graded exercise test on cycle ergometer; 50 W start, +35 W every 3 min; volitional exhaustion (substrate oxidation analyzed at 50/85/120 W)	Astaxanthin 6 mg/day, 1 capsule/day, 4 weeks; 1-week washout; placebo matched	Fat oxidation rate, carbohydrate oxidation rate, RER (during GXT stages)	GSH, H_2_O_2_, AOPP, MDA, TAG, total cholesterol	GSH ↑ vs. placebo (*p* = 0.02); H_2_O_2_ NS; MDA NS; AOPP NS; fat oxidation NS across treatments (*p* > 0.05)
Barker (2023) [41]	USA	Resistance-trained men	10/9	22.6 ± 2.2	100.0%	Eccentric leg press	Astaxanthin 12 mg/day × 4 weeks	1RM and reps-to-failure (65–75% 1RM): no change	↓ DOMS (SORE, *p* = 0.01); ↓ VAS (*p* = 0.009); PRS, SRPE no change	AX reduced subjective soreness without affecting strength or exertion
Nieman (2023) [42]	USA	Healthy trained runners	18 (cross-over)	42.2 ± 2.8	61.0%	2.25 h run at 70% VO_2_max	Astaxanthin 8 mg/day × 4 weeks	No change in performance metrics	↑ IgM recovery; no change in IL-6, IL-8, IL-10, CK, oxylipins	AX restored immune proteins and IgM post-exercise; no impact on inflammation or performance
Waldman (2023) [8]	USA	Resistance-trained males	13 (cross-over)	23.4 ± 2.1	100.0%	Drop-jump EIMD (100 jumps × 0.6 m)	Astaxanthin 12 mg/day × 4 weeks	↓ VO_2_ (*p* = 0.02); no change in Wingate power	No change in CK, IL-6, CRP, AOPP, DOMS; ↓ insulin (–24%, *p* = 0.05); ↓ SBP (–7%, *p* = 0.04)	AX had no effect on inflammation or DOMS, but improved cardiometabolic indicators (SBP, insulin, VO_2_)
Gonzalez (2024) [43]	USA	Healthy career firefighters	15(crossover)	34.5 ± 7.4	100%	Incremental maximal treadmill CPXT to fatigue; Fire Ground Test (FGT, 9 tasks)	Astaxanthin 12 mg/day (2 capsules/day) vs. placebo; 4 weeks per phase; 2-week washout	VO_2_peak (absolute/relative) NS; TTE NS; VANT (L/min) ↑ (trend); VANT (%VO_2_peak) ↑; FGT time NS	Blood cytokines (e.g., IL-1β, TNF-α, etc.) attenuated vs. baseline mainly in PLA; saliva IL-1β/cortisol/uric acid attenuated (mostly within-group); fasting oxidative stress markers (AOPP, AGEs, adiponectin) NS; blood lipids NS	AX increased VANT (%VO_2_peak) vs. PLA; no effect on VO_2_peak, TTE, substrate oxidation, or FGT performance; limited evidence of blunted exercise/FGT inflammatory-stress responses
Liu (2024) [44]	China	Elite rugby players	HBO + AST: n = 10; HBO: n = 5; Control: n = 5	21.1 ± 1.7	50%	Incremental cycling to exhaustion (7 stages)	Astaxanthin 16 mg post-exercise + HBO (1.3 ATA, 60 min) vs. HBO or rest	Tlim, Pmax (fatigue confirmation only)	Bla, CK, LDH, MDA, SOD, GSH-Px; SaO_2_, SmO_2_; T/C	HBO + AST improved lactate clearance and reduced CK and MDA vs. control and HBO alone; AST-specific effect not isolated
Tsao (2025) [11]	Taiwan, China	Healthy active males	10 (cross-over)	22.5 ± 0.9	100.0%	Cycling to exhaustion 75% VO_2_max	Astaxanthin 28 mg/day × 4 days	↑ TTE (+18%, *p* < 0.001)	↓ CK (*p* = 0.023), ↓ LDH (*p* = 0.004), ↓ MDA (*p* = 0.021); TNF-α and CRP NS	Short-term high-dose AX enhanced endurance and reduced oxidative muscle damage
Wang (2025) [45]	China	Moderately-trained healthy adults	6 mg: n = 8; 12 mg: n = 8; 24 mg: n = 8; placebo: n = 8	22.3 ± 2.1	100.0%	Incremental cycling to exhaustion (workloads at 60–85% VO_2_peak)	Astaxanthin 6/12/24 mg·d^−1^ vs. placebo, 4 weeks	Knee extension peak torque (60°/s, 180°/s); vertical jump (no VO_2_max/TTE as outcomes)	MDA, SOD, TNF-α, IL-6, CK, LDH; VAS soreness	12 mg improved CK (↓), VAS (↓), and knee extension peak torque at 180°/s (↑) vs. placebo; 12 and 24 mg improved MDA (↓)/SOD (↑) and TNF-α (↓); 6 mg showed no clear effect.
Zhang (2025) [46]	China	Young male Taekwondo athletes	18/19	16.38 ± 0.98	100%	20 s double jump kick test; 60 s high roundhouse kick test; regular Taekwondo training (5 sessions/week, ~120 min/session)	Astaxanthin 12 mg/day, once daily (morning), 4 weeks	Double jump kick (kicks/20 s) ↑; high roundhouse kick (kicks/60 s) ↑	NR	Improved sport-specific kicking performance vs. placebo in weeks 1–4 (double jump kick) and weeks 2–4 (high roundhouse); body composition NS; no adverse events reported

Abbreviations: AS, astaxanthin; CON, control; EXP, experimental; HBO, hyperbaric oxygen; VO_2_max, maximal oxygen uptake; VO_2_peak, peak oxygen uptake; TTE, time to exhaustion; TT, time trial; PP, peak power; MP, mean power; 1RM, one-repetition maximum; HIIT, high-intensity interval training; CK, creatine kinase; LDH, lactate dehydrogenase; CRP, C-reactive protein; IL-6, interleukin-6; TNF-α, tumor necrosis factor-alpha; MDA, malondialdehyde; TAC, total antioxidant capacity; SOD, superoxide dismutase; GPx, glutathione peroxidase; ROS, reactive oxygen species; DOMS, delayed-onset muscle soreness; RPE, rating of perceived exertion; HR, heart rate; HRmax, maximal heart rate; BMI, body mass index; BW, body weight; BM, body mass; SD, standard deviation; CI, confidence interval; NR, not reported; NA, not available; wk, weeks; d, days; mg·day^−1^, milligrams per day; mL·kg^−1^·min^−1^, milliliters per kilogram per minute; ↓, decrease/reduction; ↑, increase/improvement; ↑↑, marked increase.

## Data Availability

All data generated or analyzed during this study are included in this published article and its Supplementary Materials. The datasets used or analyzed during the current study are available from the corresponding author upon reasonable request.

## Supplementary Materials

The following supporting information can be downloaded at: https://www.mdpi.com/article/10.3390/nu18101570/s1, Section S1: search strategies for PubMed, Cochrane Library, Embase, Web of Science, EBSCOhost, and CNKI; Section S2 (Figure S1): risk-of-bias summary of the included randomized controlled trials; Section S3 (Figure S2A–D): distribution plots for IL-6, MDA, time-trial performance, and maximal workload/power-related outcomes; Section S4 (Figure S3A–E): precision plots for IL-6, MDA, time-trial performance, maximal workload/power-related outcomes, and VO_2_max; and Section S5: PRISMA 2020 checklist.
