# Supplementary material for: The Effects of Astaxanthin Supplementation on Exercise Recovery Biomarkers and Exercise Performance: A Systematic Review and Meta-Analysis
Source: Nutrients. 2026 May 15;18(10):1570. doi: 10.3390/nu18101570 (PMC13210138; doi:10.3390/nu18101570)
Supplement: Supplementary file 1 [file nutrients-18-01570-s001.zip › nutrients-4274258-supplementary.pdf]

Supplementary materials	Page
**Section S1.** Search strategy of PubMed, Cochrane Library, Embase, Web of Science, EBSCOhost,and CNKI.	2-3
**Section S2.** Figure S1. Risk of bias summary of the included randomized controlled trials.	4
**Section S3.** Figures S2A–S2D. Distribution plots for IL-6, MDA, TT, and WRmax.	5-6
**Section S4.** Figures S3A–S3E. Precision plots for IL-6, MDA, TT, WRmax, and VO_2max.	7-9
**Section S5.** PRISMA 2020 checklist	16-21

Section S1. Search strategy of PubMed, Cochrane Library, Embase, Web of Science, EBSCOhost, and CNKI

PubMed 226

(astaxanthin OR "Haematococcus pluvialis" OR haematococcus OR "Haematococcus") AND ("Exercise"[Mesh] OR "Physical Fitness"[Mesh] OR "Sports"[Mesh] OR exercise OR training OR sport* OR athlete* OR "physical activity" OR endurance OR aerobic OR anaerobic OR resistance OR strength OR "high-intensity" OR HIIT OR "interval training" OR "performance test" OR "exercise test") AND ("Physical Endurance"[Mesh] OR "Exercise Tolerance"[Mesh] OR "Recovery of Function"[Mesh] OR "Muscle Fatigue"[Mesh] OR "Muscle Strength"[Mesh] OR "Biomarkers"[Mesh] OR performance OR "exercise performance" OR "endurance performance" OR capacity OR fitness OR recovery OR "post-exercise recovery" OR fatigue OR soreness OR DOMS OR "delayed onset muscle soreness" OR "VO2max" OR "maximal oxygen uptake" OR "time to exhaustion" OR "time trial" OR power OR output OR CK OR "creatine kinase" OR LDH OR CRP OR "C-reactive protein" OR IL-6 OR TNF OR cytokine* OR inflammation OR oxidative OR "oxidative stress" OR antioxidant*) AND ("Randomized Controlled Trial"[Publication Type] OR "Controlled Clinical Trial"[Publication Type] OR randomized OR randomised OR placebo OR control* OR crossover OR "cross-over" OR blinded OR double-blind OR single-blind OR trial)

Web of Science 296

TS=(astaxanthin OR "Haematococcus pluvialis" OR haematococcus OR Haematococcus)AND

TS=("physical activity" OR exercise OR training OR sport* OR athlete* OR endurance OR aerobic OR anaerobic OR resistance OR strength OR "high-intensity" OR HIIT OR "interval training" OR "performance test" OR "exercise test")AND TS=(performance OR "exercise performance" OR "endurance performance" OR capacity OR fitness OR recovery OR "post-exercise recovery" OR fatigue OR soreness OR DOMS OR "delayed onset muscle soreness" OR VO2max OR "maximal oxygen uptake" OR "time to exhaustion" OR "time trial" OR power OR output OR CK OR "creatine kinase" OR LDH OR CRP OR "C-reactive protein" OR IL-6 OR TNF OR cytokine* OR inflammation OR oxidative OR "oxidative stress" OR antioxidant*)

AND TS=(randomized OR randomised OR placebo OR control* OR crossover OR "cross-over" OR blinded OR "double-blind" OR "single-blind" OR trial)

Embase 168

('astaxanthin'/exp OR astaxanthin:ti,ab,kw OR 'haematococcus pluvialis'/exp OR 'haematococcus':ti,ab,kw)

AND('exercise'/exp OR 'physical activity'/exp OR 'sport'/exp OR exercise:ti,ab,kw OR training:ti,ab,kw OR sport*:ti,ab,kw OR athlete*:ti,ab,kw OR endurance:ti,ab,kw OR aerobic:ti,ab,kw OR anaerobic:ti,ab,kw OR resistance:ti,ab,kw OR strength:ti,ab,kw OR 'high intensity interval training'/exp OR HIIT:ti,ab,kw OR 'interval training':ti,ab,kw OR 'exercise test'/exp)

AND(performance:ti,ab,kw OR 'exercise performance':ti,ab,kw OR 'endurance performance':ti,ab,kw OR capacity:ti,ab,kw OR fitness:ti,ab,kw OR recovery:ti,ab,kw OR fatigue:ti,ab,kw OR soreness:ti,ab,kw OR DOMS:ti,ab,kw OR 'delayed onset muscle soreness':ti,ab,kw OR VO2max:ti,ab,kw OR 'maximal oxygen uptake':ti,ab,kw OR 'time to exhaustion':ti,ab,kw OR 'time trial':ti,ab,kw OR power:ti,ab,kw OR output:ti,ab,kw OR CK:ti,ab,kw OR 'creatine kinase'/exp OR LDH:ti,ab,kw OR CRP:ti,ab,kw OR 'C reactive protein'/exp OR IL-6:ti,ab,kw OR TNF:ti,ab,kw OR cytokine*:ti,ab,kw OR inflammation:ti,ab,kw OR oxidative:ti,ab,kw OR 'oxidative stress'/exp OR antioxidant*:ti,ab,kw)AND

('randomized controlled trial'/exp OR randomized:ti,ab,kw OR randomised:ti,ab,kw OR placebo:ti,ab,kw

OR control*:ti,ab,kw OR crossover:ti,ab,kw OR 'cross-over':ti,ab,kw OR blinded:ti,ab,kw

OR 'double blind'/exp OR 'single blind'/exp OR trial:ti,ab,kw)

EBSCO 151

((TI(astaxanthin) OR AB(astaxanthin) OR KW(astaxanthin) OR "Haematococcus pluvialis" OR haematococcus OR Haematococcus))AND((TI(exercise OR training OR sport* OR athlete* OR "physical activity") OR AB(exercise OR training OR sport* OR athlete* OR endurance OR aerobic OR anaerobic OR resistance OR strength OR "high-intensity" OR HIIT OR "interval training" OR "exercise test" OR "performance test") OR SU(exercise OR sport)))AND((performance OR "exercise performance" OR "endurance performance" OR recovery OR fatigue OR soreness OR DOMS OR "delayed onset muscle soreness" OR VO2max OR "maximal oxygen uptake" OR "time to exhaustion" OR "time trial" OR CK OR "creatine kinase" OR LDH OR CRP OR IL-6 OR TNF OR cytokine* OR inflammation OR oxidative OR "oxidative stress" OR antioxidant*))AND((randomized OR randomised OR placebo OR control* OR crossover OR "cross-over" OR blinded OR "double-blind" OR "single-blind" OR trial))Cochrane Library

(spirulina OR arthrospira OR chlorella OR "ecklonia cava" OR fucoidan OR fucoxanthin OR "brown seaweed" OR "green algae" OR microalgae OR astaxanthin OR haematococcus)

AND(exercise OR training OR sport OR athlete OR endurance OR aerobic OR anaerobic OR "resistance training")AND(performance OR recovery OR "VO2max" OR "time to exhaustion" OR "time trial" OR power OR fatigue OR soreness OR DOMS OR CK OR "creatine kinase" OR CRP OR "C-reactive protein" OR LDH OR inflammation OR "oxidative stress")

Cochrane Library 52

#1 (astaxanthin OR "Haematococcus pluvialis" OR haematococcus OR Haematococcus) 321

#2 (exercise OR training OR sport* OR athlete* OR "physical activity"

OR endurance OR aerobic OR anaerobic OR resistance OR strength

OR HIIT OR "interval training" OR "exercise test" OR "performance test") 375371

#3 (performance OR recovery OR fatigue OR DOMS OR VO2max

OR "time to exhaustion" OR "time trial" OR CK

OR "creatine kinase" OR LDH OR CRP OR IL-6 OR TNF

OR inflammation OR oxidative OR "oxidative stress") 385913

#4 #1 AND #2 AND #3 52

CNKI 302

(Title/Abstract/Keywords: astaxanthin OR "Haematococcus pluvialis" OR Haematococcus*)

AND(Full text: exercise OR training OR sport* OR aerobic OR anaerobic OR resistance OR strength)AND(Full text: performance OR recovery OR fatigue OR soreness OR VO2max OR CK OR LDH OR "oxidative stress")

Section S2. Figure S1. Risk-of-bias summary of the included randomized controlled trials

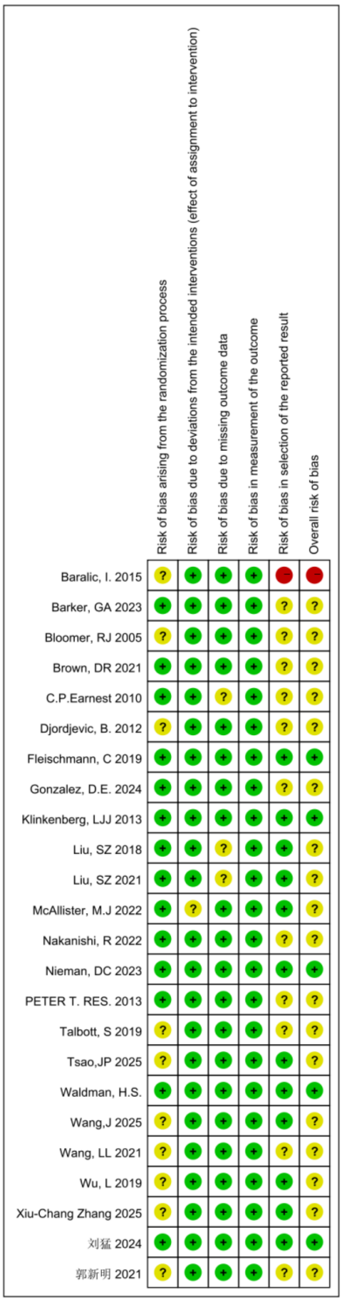

Figure S1. Risk-of-bias summary of the included randomized controlled trials assessed using RoB 2. Green indicates low risk, yellow indicates some concerns, and red indicates high risk across domains and overall judgments.

Section S3. Figures S2A–S2D. Distribution plots for IL-6, MDA, TT, and WRmax.

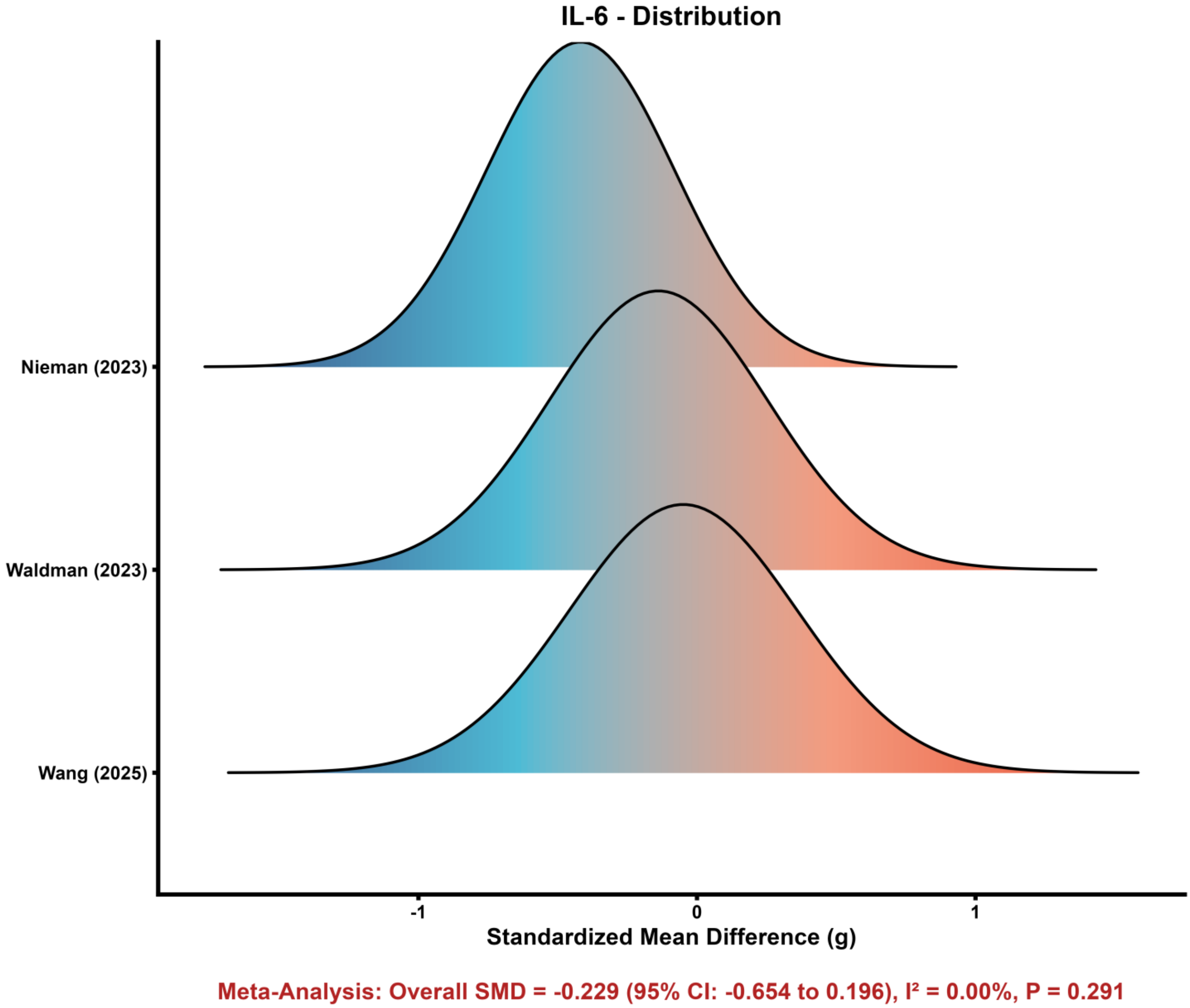

Figure S2A. Distribution plot of study-specific effect estimates for interleukin-6 (IL-6). Each point represents one comparison, and the vertical reference line indicates the null effect. This figure complements the pooled IL-6 result reported in the main text.

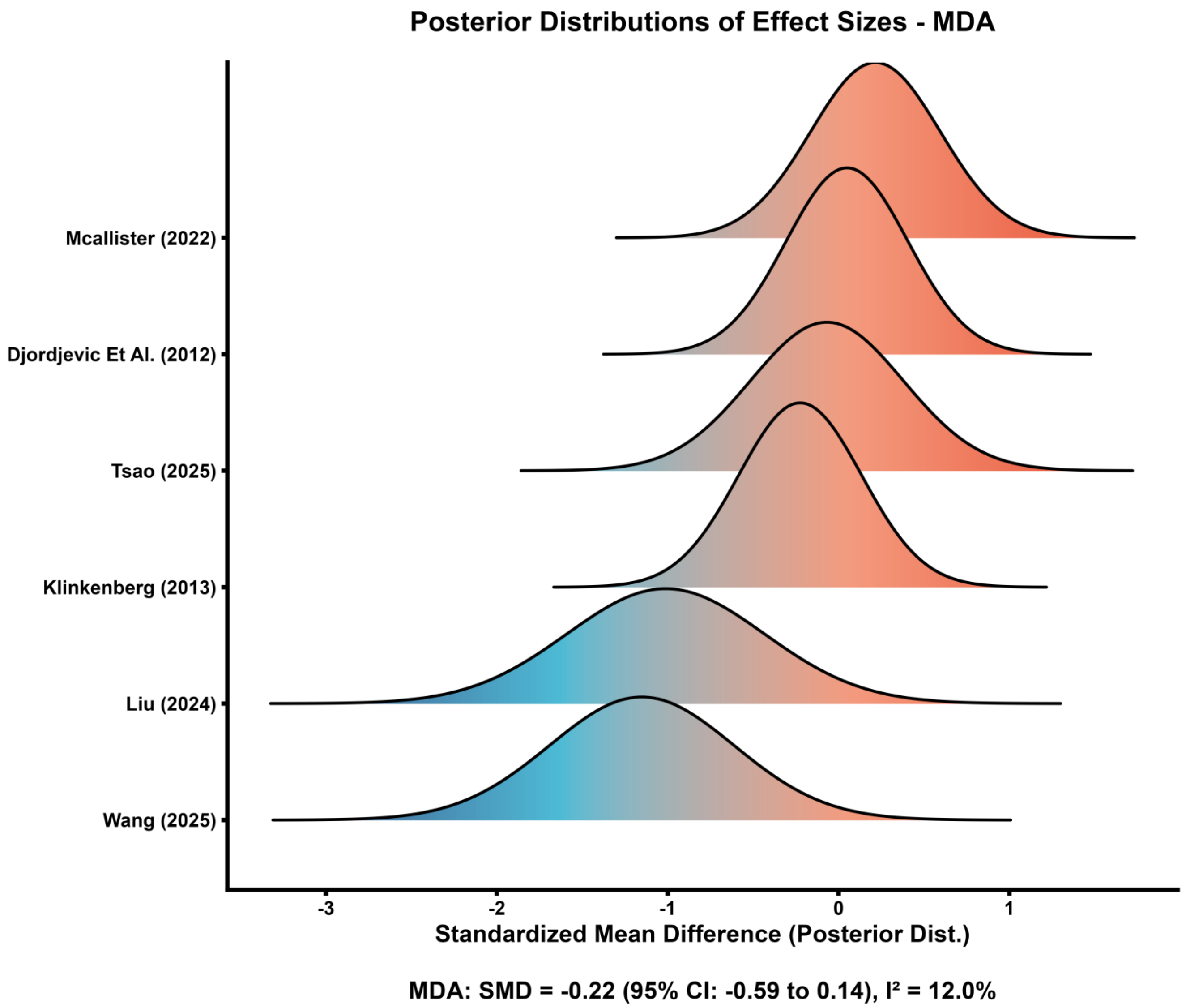

Figure S2B. Distribution plot of study-specific effect estimates for malondialdehyde (MDA). Each point represents one comparison, and the vertical reference line indicates the null effect. This figure complements the pooled MDA result reported in the main text.

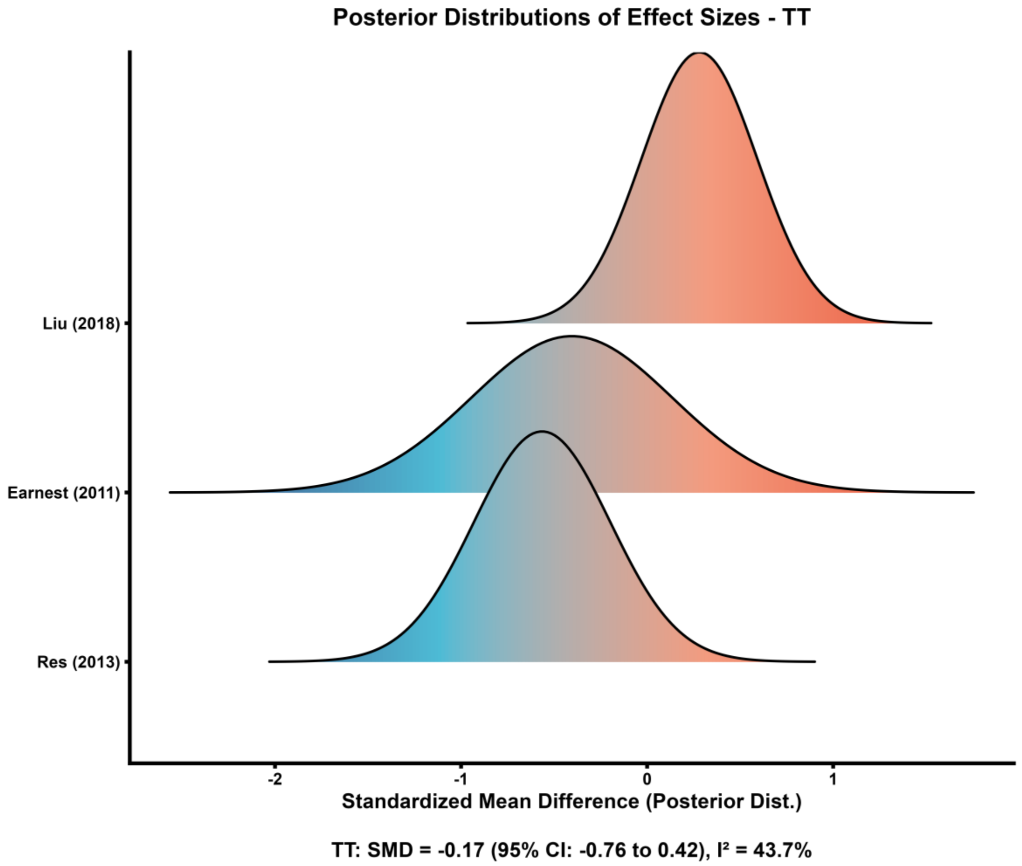

Figure S2C. Distribution plot of study-specific effect estimates for time-trial (TT) performance. Each point represents one comparison, and the spread of estimates illustrates the variability underlying the pooled TT analysis in the main text.

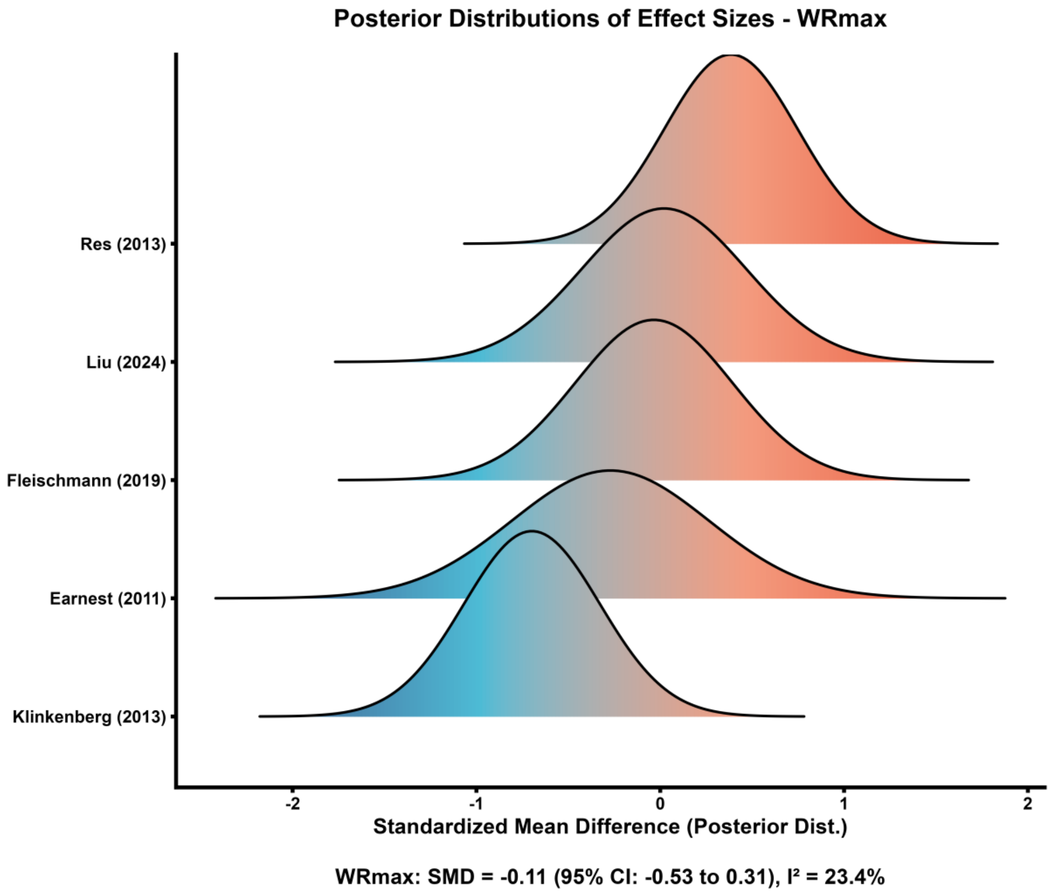

Figure S2D. Distribution plot of study-specific effect estimates for maximal workload/power-related outcomes (WRmax). Each point represents one comparison, showing the dispersion of study-specific effects contributing to the pooled workload/power-related analysis.

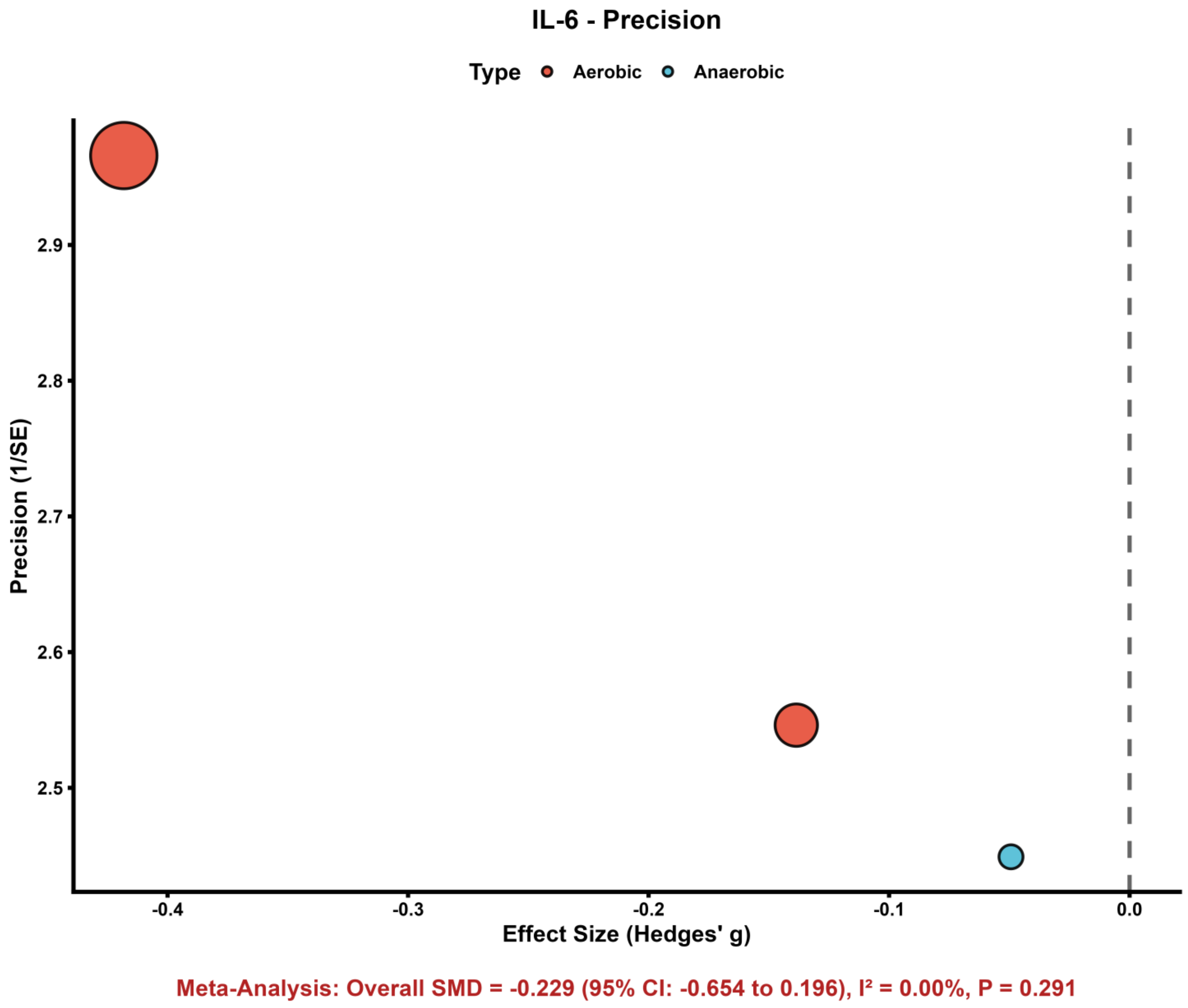

Figure S3A. Precision plot for interleukin-6 (IL-6). Each circle represents one comparison; the x-axis shows the effect size and the y-axis shows statistical precision (1/SE). This exploratory plot helps visualize the stability of the IL-6 evidence base.

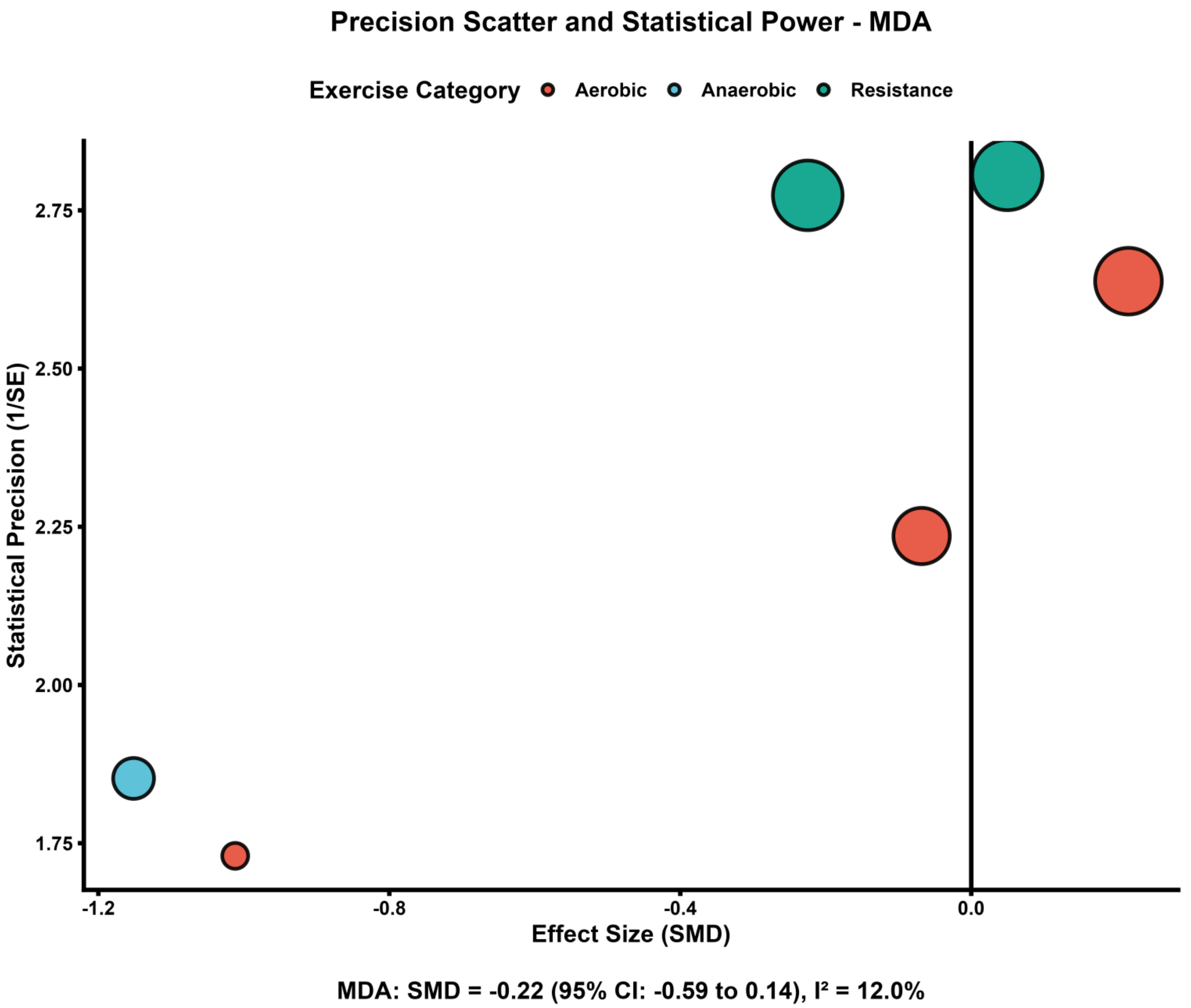

Figure S3B. Precision plot for malondialdehyde (MDA). Each circle represents one comparison; the x-axis shows the effect size and the y-axis shows statistical precision (1/SE). This figure complements the pooled MDA analysis reported in the main text.

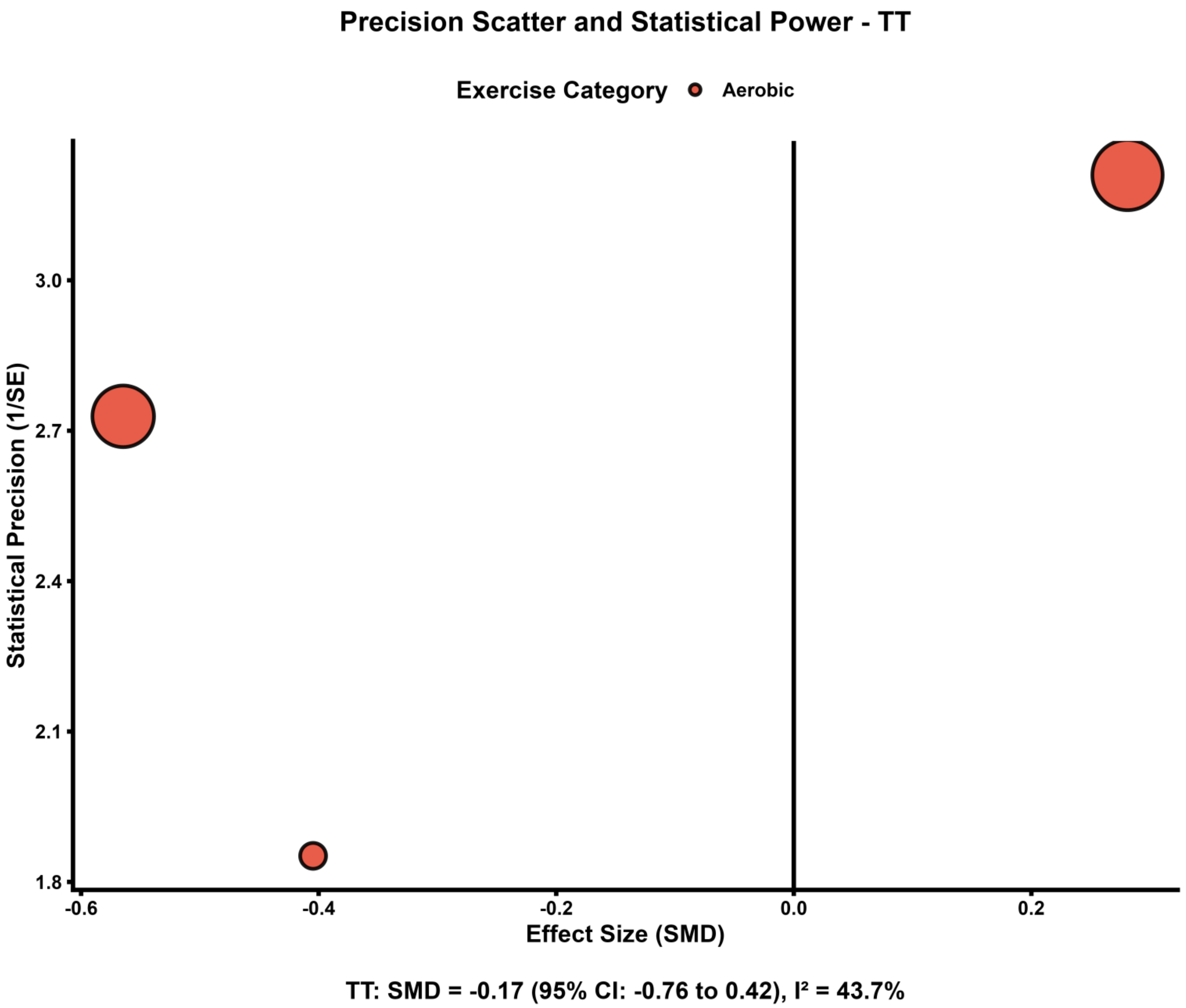

Figure S3C. Precision plot for time-trial (TT) performance. Each circle represents one comparison; the x-axis shows the effect size and the y-axis shows statistical precision (1/SE). The plot highlights the limited and heterogeneous TT evidence base.

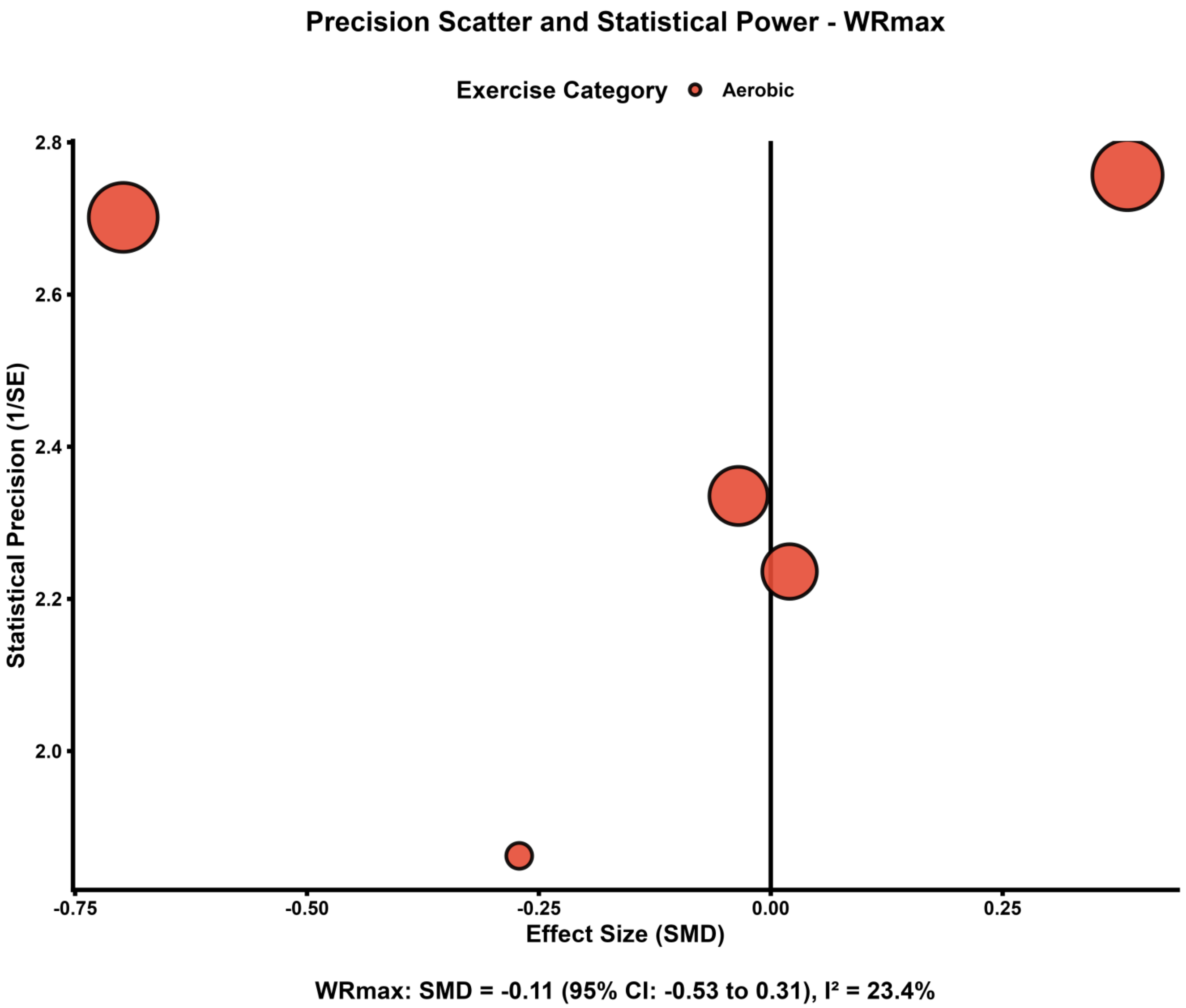

Figure S3D. Precision plot for maximal workload/power-related outcomes (WRmax). Each circle represents one comparison; the x-axis shows the effect size and the y-axis shows statistical precision (1/SE). The dispersion of circles is consistent with the pooled WRmax analysis in the main text.

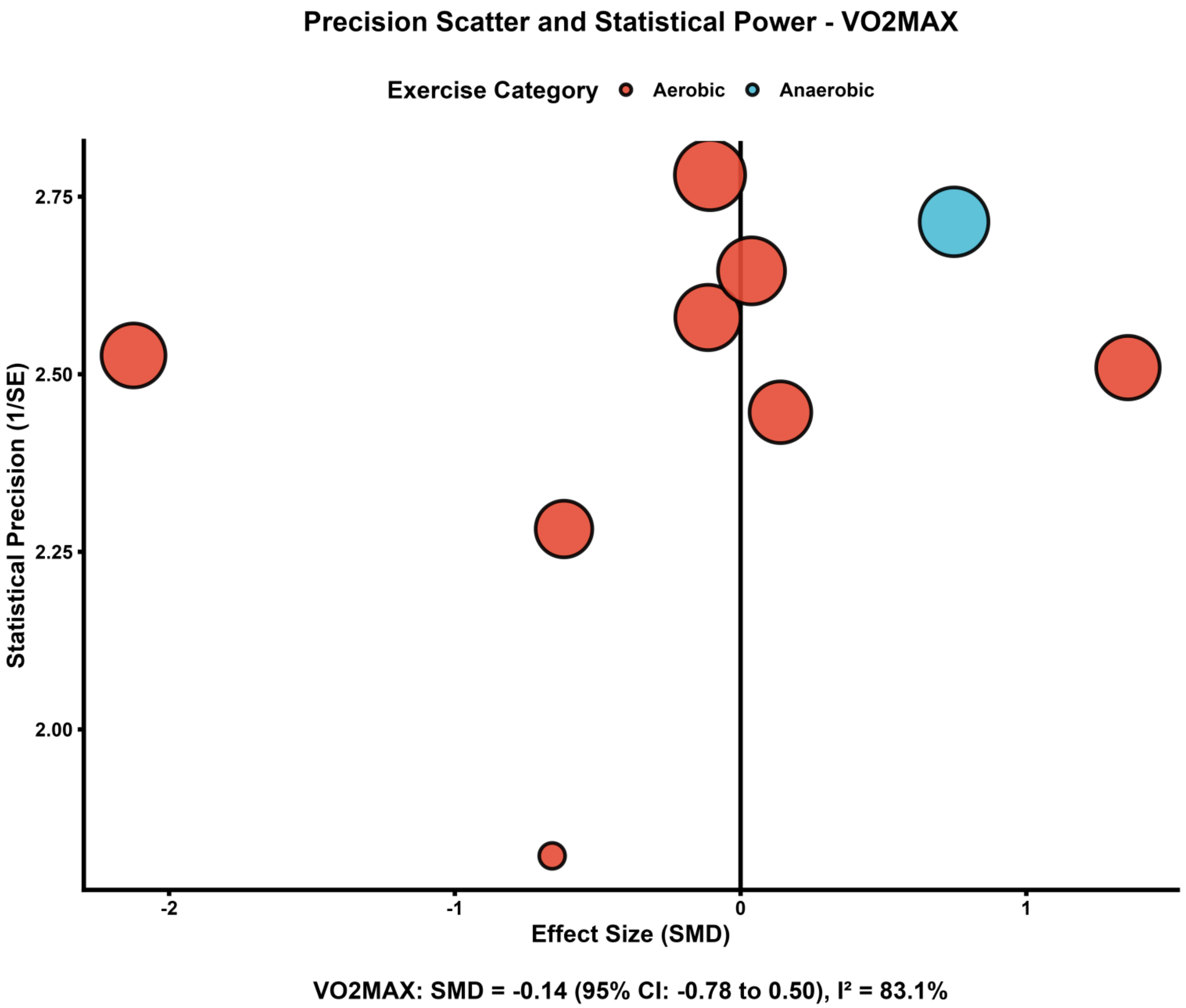

Figure S3E. Precision plot for VO_2max. Each circle represents one comparison; the x-axis shows the effect size and the y-axis shows statistical precision (1/SE). This exploratory plot illustrates the broad spread of study-specific estimates underlying the heterogeneous VO_2max result.

Section S5. PRISMA 2020 checklist

Section and Topic	Item #	Checklist item	Location where item is reported
Title	1	Identify the report as a systematic review.	Yes. Title page: "The Effects of Astaxanthin Supplementation on Exercise Recovery Biomarkers and Exercise Performance: A Systematic Review and Meta-Analysis".
Abstract	2	See the PRISMA 2020 for Abstracts checklist.	Yes. Structured abstract includes Background, Methods, Results, and Conclusions.
Rationale	3	Describe the rationale for the review in the context of existing knowledge.	Yes. Introduction, paragraphs 1–5: summarizes exercise-induced physiological stress, the biological plausibility of astaxanthin, inconsistent human trial findings, the distinction between recovery biomarkers and performance outcomes, and the rationale for the present review.
Objectives	4	Provide an explicit statement of the objective(s) or question(s) the review addresses.	Yes. Final paragraph of the Introduction: explicitly states the aim to evaluate the effects of astaxanthin supplementation on exercise recovery biomarkers and exercise performance in healthy participants and athletic populations.
Eligibility criteria	5	Specify the inclusion and exclusion criteria for the review and how studies were grouped for the syntheses.	Yes. Section 2.3 Eligibility criteria: defines the PICOS-based inclusion and exclusion criteria, including population, intervention, comparators, outcomes, and eligible study designs, and explains how studies were grouped for the syntheses.

Section and Topic	Item #	Checklist item	Location where item is reported
Information sources	6	Specify all databases, registers, websites, organisations, reference lists and other sources searched or consulted to identify studies. Specify the date when each source was last searched or consulted.	Yes. Section 2.2 Information sources and search strategy: specifies PubMed/MEDLINE, Web of Science Core Collection, Embase, EBSCOhost (SPORTDiscus), the Cochrane Library, and CNKI, as well as trial registries, citation tracking, and conference proceedings. Last search date: January 2026.
Search strategy	7	Present the full search strategies for all databases, registers and websites, including any filters and limits used.	Yes. Section 2.2 describes the search approach, and the full database-specific search strategies are presented in Supplementary Section S1.
Selection process	8	Specify the methods used to decide whether a study met the inclusion criteria of the review, including how many reviewers screened each record and each report retrieved, whether they worked independently, and if applicable, details of automation tools used in the process.	Yes. Section 2.4 Study selection: two reviewers independently screened titles/abstracts and full texts using predefined criteria; disagreements were resolved by discussion and, when necessary, by a third reviewer. EndNote 2025 was used for deduplication.
Data collection process	9	Specify the methods used to collect data from reports, including how many reviewers collected data from each report, whether they worked independently, any processes for obtaining or confirming data from study investigators, and if applicable, details of automation tools used in the process.	Yes. Section 2.5 Data extraction: two reviewers independently extracted data using a pre-piloted form; authors were contacted when key information was missing; GetData Graph Digitizer was used for data available only in figures.
Data items	10a	List and define all outcomes for which data were sought. Specify whether all results that were compatible with each outcome domain in each study were sought (e.g. for all measures, time points, analyses), and if not, the methods used to decide which results to collect.	Yes. Sections 2.3 and 2.5: defines the performance and recovery outcomes of interest and explains how multiple outcomes, timepoints, and measures of the same construct were prioritized.
	10b	List and define all other variables for which data were sought (e.g. participant and intervention characteristics, funding sources). Describe any assumptions made about any missing or unclear information.	Yes. Section 2.5: lists extracted study characteristics, participant characteristics, intervention details, exercise protocols, and outcome variables, and describes assumptions for missing or unclear

Section and Topic	Item #	Checklist item	Location where item is reported
			data (e.g., SD imputation with assumed within-study correlations).
Study risk of bias assessment	11	Specify the methods used to assess risk of bias in the included studies, including details of the tool(s) used, how many reviewers assessed each study and whether they worked independently, and if applicable, details of automation tools used in the process.	Yes. Section 2.6 Risk of bias assessment: Cochrane RoB 2 was used by two independent reviewers, with disagreements resolved through discussion and third-reviewer adjudication when required.
Effect measures	12	Specify for each outcome the effect measure(s) (e.g. risk ratio, mean difference) used in the synthesis or presentation of results.	Yes. Section 2.7 Statistical analysis: mean differences (MDs) were used when outcomes were reported on a common scale; otherwise, standardized mean differences (SMDs; Hedges' g) with 95% confidence intervals were used.
Synthesis methods	13a	Describe the processes used to decide which studies were eligible for each synthesis (e.g. tabulating the study intervention characteristics and comparing against the planned groups for each synthesis (item #5)).	Yes. Sections 2.3, 2.5, and 2.7: studies were synthesized outcome by outcome according to eligibility, outcome structure, and interpretability.
	13b	Describe any methods required to prepare the data for presentation or synthesis, such as handling of missing summary statistics, or data conversions.	Yes. Section 2.5: describes data preparation procedures, including digitization from figures, conversion of SE/CI/p values to SDs, unit harmonization, and calculation of missing change-score SDs.
	13c	Describe any methods used to tabulate or visually display results of individual studies and syntheses.	Yes. Sections 2.5 and 2.11: results were displayed using flow diagrams, risk-of-bias figures, distribution plots, precision plots, subgroup plots, funnel plots, and Summary of Findings tables.
	13d	Describe any methods used to synthesize results and provide a rationale for the choice(s). If meta-analysis was performed, describe the model(s), method(s) to identify the presence and extent of statistical heterogeneity, and software package(s) used.	Yes. Section 2.7 Statistical analysis: frequentist random-effects meta-analysis was performed using restricted maximum likelihood (REML) estimation with the Hartung–Knapp–Sidik–Jonkman

Section and Topic	Item #	Checklist item	Location where item is reported
			adjustment. Heterogeneity was assessed using I^2 and related statistics.
	13e	Describe any methods used to explore possible causes of heterogeneity among study results (e.g. subgroup analysis, meta-regression).	Yes. Section 2.8 Subgroup and sensitivity analyses: pre-specified subgroup analyses were conducted by training status, astaxanthin dose, and intervention duration where data permitted. Formal meta-regression was planned but not performed because too few studies contributed to the pooled outcomes.
	13f	Describe any sensitivity analyses conducted to assess robustness of the synthesized results.	Yes. Section 2.8: sensitivity analyses included leave-one-out analyses, exclusion of high-risk studies, exclusion of crossover trials, alternative assumed correlations, exclusion of non-placebo-controlled studies, exclusion of studies with unclear astaxanthin source, and restriction to change-score studies where feasible.
Reporting bias assessment	14	Describe any methods used to assess risk of bias due to missing results in a synthesis (arising from reporting biases).	Yes. Section 2.9 Publication bias: funnel plots and Egger's regression test were used as exploratory assessments of publication bias and small-study effects, interpreted cautiously given the limited number of studies.
Certainty assessment	15	Describe any methods used to assess certainty (or confidence) in the body of evidence for an outcome.	Yes. Section 2.10 Certainty of evidence: certainty of evidence for pre-specified primary outcomes was assessed using the GRADE approach across the domains of risk of bias, inconsistency, indirectness, imprecision, and publication bias.

Section and Topic	Item #	Checklist item	Location where item is reported
Study selection	16a	Describe the results of the search and selection process, from the number of records identified in the search to the number of studies included in the review, ideally using a flow diagram.	Yes. Section 3.1 Study selection and Figure 1 report the number of records identified, screened, excluded, and included.
	16b	Cite studies that might appear to meet the inclusion criteria, but which were excluded, and explain why they were excluded.	Partially. Section 3.1 reports the number of full-text articles excluded and summarizes the reasons for exclusion, but excluded studies are not individually cited in the main text.
Study characteristics	17	Cite each included study and present its characteristics.	Yes. Section 3.2 Study characteristics and Table 1 present the characteristics of the included studies.
Risk of bias in studies	18	Present assessments of risk of bias for each included study.	Yes. Section 3.3 Risk of bias, Figure 2, and Supplementary Figure S1 report the risk-of-bias assessments.
Results of individual studies	19	For all outcomes, present, for each study: (a) summary statistics for each group (where appropriate) and (b) an effect estimate and its precision (e.g. confidence/credible interval), ideally using structured tables or plots.	Yes. Main-text and supplementary plots present study-specific effect estimates and their precision for the synthesized outcomes, and Table 1 summarizes study-level findings.
Results of syntheses	20a	For each synthesis, briefly summarise the characteristics and risk of bias among contributing studies.	Yes. Sections 3.4–3.5 summarize the characteristics and coherence of the contributing studies for each synthesis.
	20b	Present results of all statistical syntheses conducted. If meta-analysis was done, present for each the summary estimate and its precision (e.g. confidence/credible interval) and measures of statistical heterogeneity. If comparing groups, describe the direction of the effect.	Yes. Sections 3.4–3.5 report pooled effect estimates, 95% confidence intervals, and heterogeneity statistics for each outcome.
	20c	Present results of all investigations of possible causes of heterogeneity among study results.	Yes. Section 3.6 Subgroup analyses presents investigations of possible causes of heterogeneity.
	20d	Present results of all sensitivity analyses conducted to assess the robustness of the synthesized results.	Yes. Section 3.8 Sensitivity analyses reports leave-one-out sensitivity analyses for outcomes with substantial heterogeneity.

Section and Topic	Item #	Checklist item	Location where item is reported
Reporting biases	21	Present assessments of risk of bias due to missing results (arising from reporting biases) for each synthesis assessed.	Yes. Section 3.9 Publication bias presents exploratory funnel-plot-based assessments for selected outcomes.
Certainty of evidence	22	Present assessments of certainty (or confidence) in the body of evidence for each outcome assessed.	Yes. Section 3.10 Certainty of evidence and the GRADE Summary of Findings table present certainty assessments for the key outcomes.
Discussion	23a	Provide a general interpretation of the results in the context of other evidence.	Yes. Section 4.1 Principal findings provides a general interpretation of the results in the context of the evidence base.
	23b	Discuss any limitations of the evidence included in the review.	Yes. Sections 4.2–4.6 discuss limitations of the included evidence, including heterogeneity, small numbers of studies, and uncertainty in some pooled outcomes.
	23c	Discuss any limitations of the review processes used.	Yes. Section 4.6 Implications, strengths, and limitations discusses limitations of the review process, including methodological heterogeneity and the role of narrative-only evidence.
	23d	Discuss implications of the results for practice, policy, and future research.	Yes. Sections 4.4–4.6 and the Conclusion discuss implications for practice and future research.
Registration and protocol	24a	Provide registration information for the review, including register name and registration number, or state that the review was not registered.	Yes. Section 2.1 Study design and reporting standards: protocol registered in PROSPERO (CRD420251119762).
	24b	Indicate where the review protocol can be accessed, or state that a protocol was not prepared.	Yes. Section 2.1 indicates that the protocol was prospectively registered in PROSPERO.

Section and Topic	Item #	Checklist item	Location where item is reported
	24c	Describe and explain any amendments to information provided at registration or in the protocol.	Yes. Section 2.1 states that deviations from the protocol were documented and justified in the Supplementary Materials.
Support	25	Describe sources of financial or non-financial support for the review, and the role of the funders or sponsors in the review.	Yes. Funding information is reported in the Funding statement.
Competing interests	26	Declare any competing interests of review authors.	Yes. Competing interests are reported in the Competing interests statement.
Availability of data, code and other materials	27	Report which of the following are publicly available and where they can be found: template data collection forms; data extracted from included studies; data used for all analyses; analytic code; any other materials used in the review.	Partially. The Availability of data and materials statement indicates that data are included in the article and supplementary materials, and additional materials may be available from the corresponding author upon reasonable request. The review does not explicitly provide a public repository link for extracted datasets, code, or data collection forms.
